# Insulin-regulated serine and lipid metabolism drive peripheral neuropathy

**DOI:** 10.1038/s41586-022-05637-6

**Published:** 2023-01-25

**Authors:** Michal K. Handzlik, Jivani M. Gengatharan, Katie E. Frizzi, Grace H. McGregor, Cameron Martino, Gibraan Rahman, Antonio Gonzalez, Ana M. Moreno, Courtney R. Green, Lucie S. Guernsey, Terry Lin, Patrick Tseng, Yoichiro Ideguchi, Regis J. Fallon, Amandine Chaix, Satchidananda Panda, Prashant Mali, Martina Wallace, Rob Knight, Marin L. Gantner, Nigel A. Calcutt, Christian M. Metallo

**Affiliations:** 1 Molecular and Cell Biology Laboratory, The Salk Institute for Biological Studies, La Jolla, CA USA; 2 Department of Bioengineering, University of California San Diego, La Jolla, CA USA; 3 Department of Pathology, School of Medicine, University of California San Diego, La Jolla, CA USA; 4 Department of Pediatrics, School of Medicine, University of California San Diego, La Jolla, CA USA; 5 Bioinformatics and Systems Biology Program, University of California San Diego, La Jolla, CA USA; 6 Center for Microbiome Innovation, University of California San Diego, La Jolla, CA USA; 7 Regulatory Biology Laboratory, The Salk Institute for Biological Studies, La Jolla, CA USA; 8 Scripps Research, La Jolla, CA USA; 9 Lowy Medical Research Institute, La Jolla, CA USA; 10 Department of Nutrition and Integrative Physiology, University of Utah, Salt Lake City, UT USA; 11 School of Agriculture and Food Science, University College Dublin, Dublin, Ireland

**Keywords:** Metabolic diseases, Metabolomics

## Abstract

Diabetes represents a spectrum of disease in which metabolic dysfunction damages multiple organ systems including liver, kidneys and peripheral nerves^1,2^. Although the onset and progression of these co-morbidities are linked with insulin resistance, hyperglycaemia and dyslipidaemia^3–7^, aberrant non-essential amino acid (NEAA) metabolism also contributes to the pathogenesis of diabetes^8–10^. Serine and glycine are closely related NEAAs whose levels are consistently reduced in patients with metabolic syndrome^10–14^, but the mechanistic drivers and downstream consequences of this metabotype remain unclear. Low systemic serine and glycine are also emerging as a hallmark of macular and peripheral nerve disorders, correlating with impaired visual acuity and peripheral neuropathy^15,16^. Here we demonstrate that aberrant serine homeostasis drives serine and glycine deficiencies in diabetic mice, which can be diagnosed with a serine tolerance test that quantifies serine uptake and disposal. Mimicking these metabolic alterations in young mice by dietary serine or glycine restriction together with high fat intake markedly accelerates the onset of small fibre neuropathy while reducing adiposity. Normalization of serine by dietary supplementation and mitigation of dyslipidaemia with myriocin both alleviate neuropathy in diabetic mice, linking serine-associated peripheral neuropathy to sphingolipid metabolism. These findings identify systemic serine deficiency and dyslipidaemia as novel risk factors for peripheral neuropathy that may be exploited therapeutically.

## Abstract (web summary)

Serine deficiency can increase small fibre neuropathy in wild-type mice and serine replacement in diabetic mice alleviates diabetic neuropathy, directly linking amino acid metabolism to peripheral nerve disorders.

## Main

**Fig. 1:**
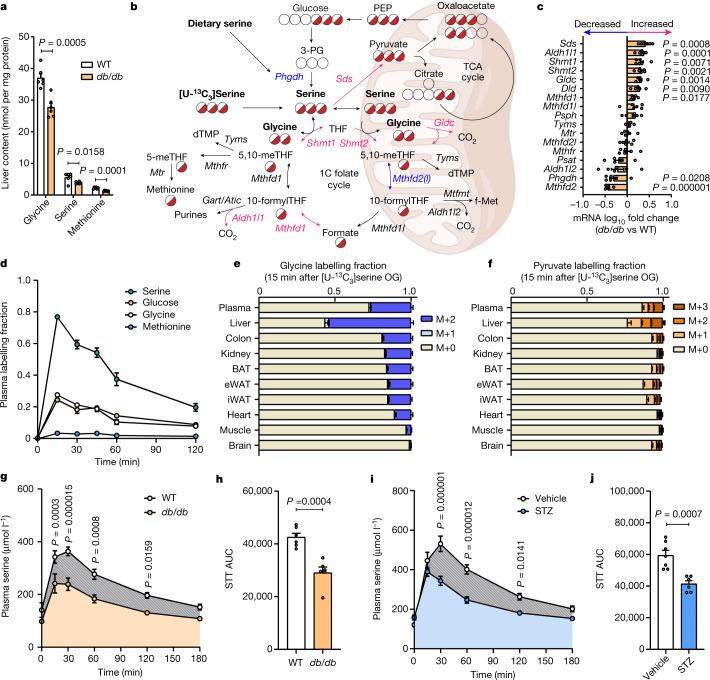
Sources and sinks of altered serine metabolism in diabetes. **a**, Levels of glycine, serine and methionine in the liver of wild-type and BKS-*db/db* mice after a 6-h fast (*n* = 6 per group). **b**, Schematic of serine and glycine biosynthetic and catabolic pathways. Upregulated hepatic genes in BKS-*db/db* mice are in purple, and downregulated are genes are in blue. 10-formylTHF, 10-formyltetrahydrofolate; 3-PG, 3-phosphoglycerate; 5,10-meTHF, 5,10-methylenetetrahydrofolate; dTMP, deoxythymidine monophosphate; f-Met, *N*-formylmethionine; PEP, phosphoenol pyruvate; TCA, tricarboxylic acid; THF, tetrahydrofolate. **c**, mRNA expression of liver enzyme genes regulating SGOC metabolism in wild-type and BKS-*db/db* mice (*n* = 6 per group). **d**, Plasma serine, glucose, glycine and methionine-labelling fraction (1 − M_0_) in wild-type mice administered [U-^13^C_3_]serine via oral gavage after an overnight fast (*n* = 4 per time point). **e**, Tissue glycine labelling fraction in wild-type mice 15 min after [U-^13^C_3_]serine administration via oral gavage (*n* = 4 per tissue) following an overnight fast. **f**, Tissue pyruvate labelling fraction in wild-type mice 15 min after [U-^13^C_3_]serine administration via oral gavage (*n* = 4 per tissue) after an overnight fast. **g**, Combined OGTT and STT in wild-type and BKS-*db/db* mice (*n* = 6 per group) after an overnight fast. **h**, STT AUC in wild-type and BKS-*db/db* mice (*n* = 6 per group). **i**, Combined OGTT and STT in vehicle- (*n* = 7) and STZ-treated (*n* = 6) C57BL/6J mice after an overnight fast. **j**, STT AUC in vehicle- (*n* = 7) and STZ-treated (*n* = 6) C57BL/6J mice. Data are mean ± s.e.m., and were analysed using two-sided independent *t*-test (**a**,**c**,**h**,**j**) and two-way ANOVA with Fisher’s least significant difference post hoc test (**g**,**i**). The schematic in Fig. 1b was prepared in BioRender.

**Extended Data Fig. 1:**
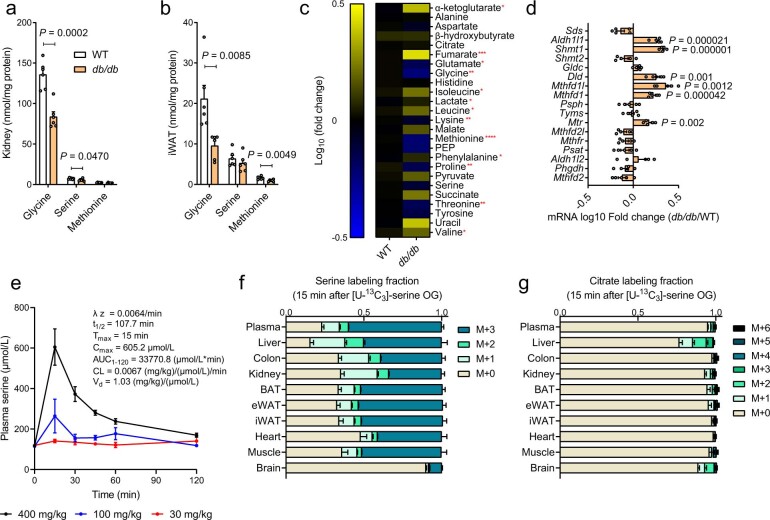
Serine and glycine metabolism in diabetes. (**a**) Levels of glycine, serine, and methionine in WT and BKS-*db/db* mice in the kidney (n = 6 per group). (**b**) Levels of glycine, serine, and methionine in WT and BKS-*db/db* mice in the inguinal adipose tissue (iWAT) (n = 6 per group). (**c**) Plasma metabolite levels in WT and BKS-*db/db* mice (n = 6 per group). (**d**) mRNA expression of kidney enzymes regulating serine, glycine, and one-carbon metabolism in WT (n = 6) and BKS-*db/db* mice (n = 5). (**e**) Serine tolerance test (STT) in C57BL/6J mice after an overnight fast (n = 4 per dose). (**f**) Tissue serine labeling fraction in WT mice 15 min after [U-^13^C_3_]serine administration via oral gavage (n = 4 per tissues) after an overnight fast. (**g**) Tissue citrate labeling fraction in WT mice 15 min after [U-^13^C_3_]serine administration via oral gavage (n = 4 per tissues) after an overnight fast. Data are mean ± standard error of mean (SEM) and were analyzed using a two-sided independent t-test (**a–d**). * *P* < 0.05, ** *P* < 0.01, *** *P* < 0.001, **** *P* < 0.0001 vs. WT group.

To explore how obesity and diabetes influence serine, glycine and one-carbon (SGOC) metabolism, we quantified serine, glycine and methionine across tissues in an established mouse model of morbid obesity, insulin resistance and hyperglycaemia (leptin receptor-deficient *db/db* mice on a black Kaliss background (BKS-*db/db*)) and compared results with age-matched wild-type C57BL/6J controls. The *db/db* mice showed reductions of around 30% in hepatic and renal serine levels relative to wild-type mice (Fig. 1a and Extended Data Fig. 1a), and the more abundant glycine pools were reduced by 30–50% in liver, kidney, inguinal white adipose tissue (iWAT) and plasma (Fig. 1a and Extended Data Fig. 1a–c). Methionine is linked to serine through one-carbon metabolism and was also reduced in liver, iWAT, and plasma (Fig. 1a and Extended Data Fig. 1b,c), suggesting that diabetes decreases serine and glycine levels in tissues that are important for glucose and lipid homeostasis.

Mammals obtain serine from the diet, de novo synthesis from glucose and via glycine and one-carbon metabolism, with the liver and kidney serving as major sites for postprandial NEAA metabolism (Fig. 1b). To better understand the mechanistic basis of reduced hepatic serine and glycine in *db/db* mice, we quantified the expression of genes associated with SGOC metabolism after a 6-h fast (Fig. 1c). Genes encoding key enzymes responsible for serine and one-carbon unit catabolism or disposal were significantly upregulated in *db/db* mice, including serine dehydratase (*Sds*), serine hydroxymethyltransferase 1 (*Shmt1*), *Shmt2* and 10-formyltetrahydrofolate dehydrogenase (*Aldh1l1*). The expression of two genes encoding components of the glycine cleavage system—glycine decarboxylase (*Gldc*) and dihydrolipoamide dehydrogenase (*Dld*)—was increased in *db/db* liver, whereas the expression of 3-phosphoglycerate dehydrogenase (*Phgdh*) and methylenetetrahydrofolate dehydrogenase 2 (*Mthfd2*) were both significantly reduced, indicating that de novo serine synthesis may also be limited in diabetic mice. These results are consistent with genome-scale metabolic modelling of transcription data from human diabetic liver^17^. The kidney is another centre for SGOC metabolism^18^ and renal expression of *Shmt1* and several genes encoding enzymes associated with one-carbon metabolism were increased in *db/db* mice (Extended Data Fig. 1d).

**Extended Data Fig. 2:**
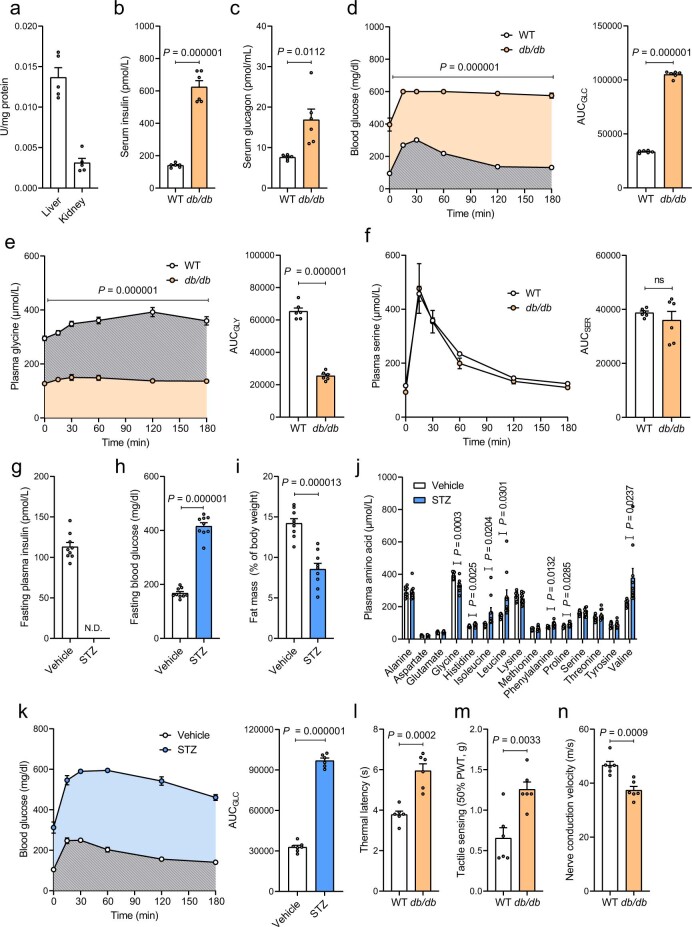
Serine tolerance test in type 1 and 2 diabetic mice. (**a**) Serine dehydratase activity determined in the liver and kidney in C57BL/6J mice in the fed state (n = 5 per tissues). (**b**) Serum insulin in WT and BKS-*db/db* mice quantified after a 6-h fast (n = 6 per group). (**c**) Serum glucagon in WT (n = 5) and BKS-*db/db* (n = 6) mice quantified after a 6-h fast. (**d**) Plasma glucose pharmacokinetics and area under curve (AUC_GLC_) in WT and BKS-*db/db* mice following GTT/STT with glucose dosed at 2 g/kg (n = 6 per group) after an overnight fast. (**e**) Plasma glycine pharmacokinetics and area under curve (AUC_GLY_) in WT and BKS-*db/db* mice following GTT/STT with glucose dosed at 2 g/kg (n = 6 per group) after an overnight fast. (**f**) Serine tolerance test (STT) and area under curve (AUC_SER_) in WT and BKS-*db/db* mice (n = 6 per group) dosed with serine (400 mg/kg) only after an overnight fast. (**g**) Fasting (6-h) plasma insulin level in C57BL/6J mice treated with vehicle or streptozotocin (STZ) 1 week after injection (n = 9 per group). (**h**) Fasting (6-h) blood glucose in C57BL/6J mice treated with vehicle or streptozotocin (STZ) 1 week after injection (n = 9 per group). (**i**) Body fat mass content in C57BL/6J mice treated with vehicle or streptozotocin (STZ) 1 week after injection (n = 9 per group). (**j**) Fasting (6-h) plasma amino acid concentration in C57BL/6J mice treated with vehicle (n = 9) or streptozotocin (STZ, n = 10) 1 week after injection. (**k**) Plasma glucose pharmacokinetics and area under curve (AUC_GLC_) after an overnight fast during GTT/STT in C57BL/6J mice treated with vehicle (n = 7) or streptozotocin (STZ, n = 6) 2 weeks after injections with glucose dosed at 2 g/kg. (**l**) Thermal latency in 14-week-old WT and BKS-*db/db* mice (n = 6 per group). (**m**) Tactile sensing in 14-week-old WT and BKS-*db/db* mice (n = 6 per group). (**n**) Motor nerve conduction velocity in 14-week-old WT and BKS-*db/db* mice (n = 6 per group). Data are mean ± standard error of mean (SEM) and were analyzed using a two-sided independent t-test (b–n) and a two-way ANOVA with Fisher’s LSD *post hoc* test (e–f, k time-course experiments).

Circadian and postprandial variations in amino acids and glucose make the diagnosis of metabolic defects challenging. We therefore hypothesized that a ‘serine tolerance test’ (STT) could better assess SGOC metabolism in mice and identify those with elevated serine disposal. Applying similar dosages to those used in human clinical trials (ClinicalTrials.gov: NCT03062449) (400 mg kg^−1^), we orally administered serine to overnight-fasted wild-type mice and quantified plasma serine pharmacokinetics to gauge the dynamics of serine clearance, with levels returning to baseline in approximately 2 h (Extended Data Fig. 1e). To identify the principal pathways involved in serine disposal, we orally administered [U-^13^C_3_]serine to overnight-fasted wild-type mice and quantified the enrichment of downstream metabolites. We observed that glucose was labelled to a similar extent as glycine throughout the test (Fig. 1d), and [U-^13^C_3_]serine-derived carbon was significantly incorporated into hepatic glycine, pyruvate and citrate pools (Fig. 1e,f and Extended Data Fig. 1f,g), demonstrating that hepatic gluconeogenesis is a major pathway for serine disposal in some contexts and linking its regulation to insulin and glucagon, which are both elevated in *db/db* mice (Extended Data Fig. 2a–c). To test whether insulin resistance affects serine absorption and disposal, we delivered both glucose (2 g kg^−1^) and serine (400 mg kg^−1^) to overnight-fasted wild-type and *db/db* mice. We observed a significant reduction in STT area under the curve (AUC) in *db/db* mice (Fig. 1g,h and Extended Data Fig. 2d), despite the higher dose administered. Notably, acute serine challenge had essentially no effect on circulating glycine concentrations (Extended Data Fig. 2e), despite evidence of their rapid interconversion (Fig. 1d,e). Conversely, there were no differences in STT AUC between wild-type and *db/db* mice when serine was administered without glucose (Extended Data Fig. 2f).

To determine whether elevated serine disposal is specific to leptin receptor-deficient *db/db* mice or is more generally attributable to impaired insulin signalling, we treated C57BL/6J mice with vehicle or streptozotocin (STZ) to induce insulin deficiency, hyperglycaemia and fat loss (Extended Data Fig. 2g–i). Plasma glycine and branched-chain amino acids (but not serine) were altered one week after STZ treatment (Extended Data Fig. 2j). Two weeks after injection, co-administration of glucose and serine revealed elevated serine disposal in STZ-diabetic mice relative to controls (Fig. 1i,j and Extended Data Fig. 2k), suggesting that insulin resistance or deficiency can both contribute to reduced circulating serine in diabetic mice.

Clinical studies have implicated serine deficiency in the regulation of macular disease and peripheral neuropathy^15,16^. Given the impaired serine homeostasis in *db/db* mice, we next confirmed that aberrant serine metabolism is coincident with peripheral neuropathy in this model. Fourteen-week-old *db/db* mice exhibited thermal and tactile hypoalgesia as well as decreased motor nerve conduction velocity (MNCV) (Extended Data Fig. 2l–n). These findings suggest that aberrant serine homeostasis is associated with diabetic peripheral neuropathy.

## Serine remodels lipid metabolism

**Fig. 2:**
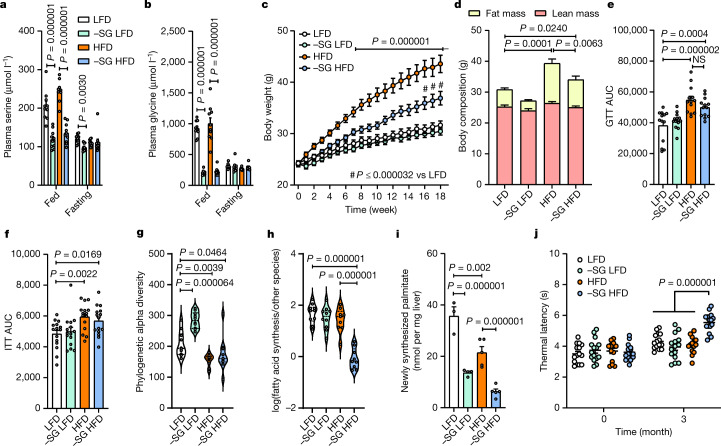
Dietary serine restriction suppresses fatty acid synthesis and mitigates adiposity. **a**, Plasma serine levels in fed and overnight-fasted mice fed with LFD, −SG LFD, HFD or −SG HFD for 18 weeks (*n* = 10 per group). **b**, Plasma glycine levels in mice fed with LFD, −SG LFD, HFD or −SG HFD for 18 weeks (*n* = 10 per group). **c**, Body weight of mice fed with LFD, −SG LFD, HFD, or −SG HFD (*n* = 13 per group). **d**, Body composition in mice 18 weeks after feeding with LFD (*n* = 10), −SG LFD (*n* = 12), HFD (*n* = 13) or −SG HFD (*n* = 13). **e**, Glucose tolerance test (GTT) AUC 18 weeks after feeding with LFD, −SG LFD, HFD or −SG HFD (*n* = 13 per group). **f**, Insulin tolerance test (ITT) AUC 18 weeks after feeding with LFD, −SG LFD, HFD or −SG HFD (*n* = 15 per group). **g**, Phylogenetic alpha diversity in mice fed with LFD, −SG LFD, HFD or −SG HFD (*n* = 10 per group). **h**, Log fraction of species from the fatty acid synthesis pathway in mice fed with LFD (*n* = 10), −SG LFD (*n* = 10), HFD (*n* = 9) or −SG HFD (*n* = 9) for 18 weeks. **i**, Hepatic de novo palmitate synthesis in mice fed with LFD (*n* = 3), −SG LFD (*n* = 4), HFD (*n* = 5) or −SG HFD (*n* = 5) for 18 weeks. **j**, Thermal sensing in mice fed with LFD (*n* = 15), −SG LFD (*n* = 15), HFD (*n* = 14) or −SG HFD (*n* = 15 per group). Data are mean ± s.e.m. and minimum and maximum (**g**,**h**), and were analysed using two-way ANOVA with Fisher’s least significant difference post hoc test (**a**–**j**).

**Extended Data Fig. 3:**
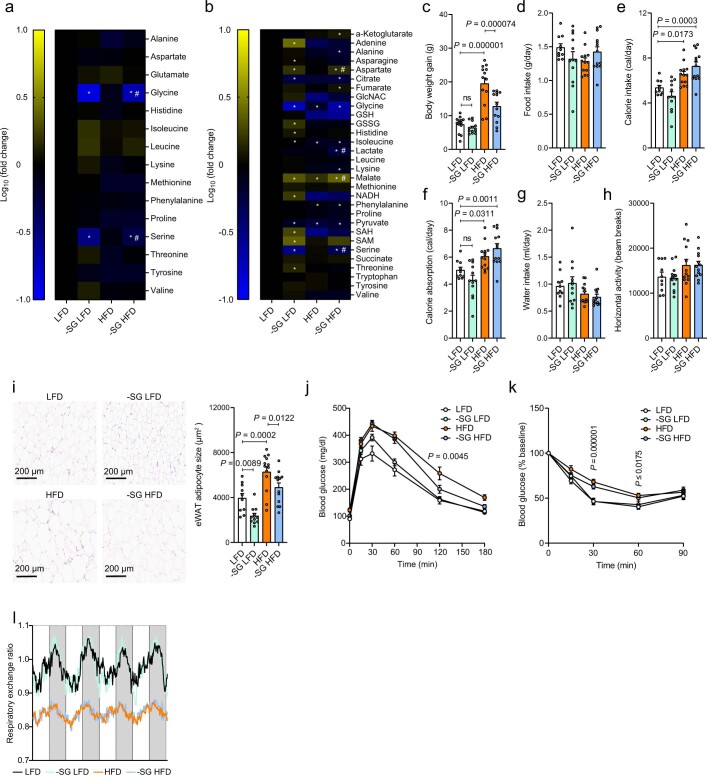
Dietary serine restriction and metabolic homeostasis. (*a*) Plasma amino acid level in the fed state in mice fed with a low fat diet (LFD, n = 5), serine/glycine-free LFD (-SG LFD, n = 8), high-fat diet (HFD, n = 8), and serine/glycine free HFD (-SG HFD, n = 8) for 18 weeks. (*b*) Liver metabolite content in mice fed with a low fat diet (LFD, n = 10), serine/glycine-free LFD (-SG LFD, n = 10), high-fat diet (HFD, n = 10), and serine/glycine free HFD (-SG HFD, n = 10) for 18 weeks. (*c*) Body weight gain 18 weeks after dietary intervention (n = 13 per group). (*d*) Food intake in response to an 18-week dietary feeding in mice with a low fat diet (LFD, n = 10), serine/glycine-free LFD (-SG LFD, n = 12), high fat diet (HFD, n = 13), and serine/glycine-free HFD (-SG HFD, n =  = 13). (*e*) Calorie intake in response to an 18-week dietary feeding in mice with a low fat diet (LFD, n = 10), serine/glycine-free LFD (-SG LFD, n = 12), high fat diet (HFD, n = 13), and serine/glycine-free HFD (-SG HFD, n = 13). (*f*) Calorie absorption in response to an 18-week dietary feeding in mice with a low fat diet (LFD, n = 10), serine/glycine-free LFD (-SG LFD, n = 12), high fat diet (HFD, n = 13), and serine/glycine-free HFD (-SG HFD, n = 13). (*g*) Water intake in response to an 18-week dietary feeding in mice with a low fat diet (LFD, n = 10), serine/glycine-free LFD (-SG LFD, n = 12), high fat diet (HFD, n = 13), and serine/glycine-free HFD (-SG HFD, n = 13). (*h*) Physical activity in response to an 18-week dietary feeding in mice with a low fat diet (LFD, n = 10), serine/glycine-free LFD (-SG LFD, n = 12), high fat diet (HFD, n = 13), and serine/glycine-free HFD (-SG HFD, n = 13). (*i*) Representative images and quantification of adipocyte size in response to an 18-week dietary feeding in mice with a low fat diet (LFD, n = 10), serine/glycine-free LFD (-SG LFD, n =  = 12), high fat diet (HFD, n = 13), and serine/glycine-free HFD (-SG HFD, n = 13). (*j*) I.P. glucose tolerance test 18 weeks after feeding with a low fat diet (LFD) or high fat diet (HFD) replete or deficient in serine and glycine (n = 13 per group). (*k*) I.P. insulin tolerance test 18 weeks after feeding with a low fat diet (LFD) or high fat diet (HFD) replete or deficient in serine and glycine (n = 15 per group). (*l*) Respiratory exchange ratio time course 18 weeks after feeding mice with a low fat diet (LFD, n = 10), serine/glycine-free LFD (-SG LFD, n = 12), high fat diet (HFD, n = 13), and serine/glycine-free HFD (-SG HFD, n =  = 13). Data are mean ± standard error of mean (SEM) and were analyzed using a two-way ANOVA with Fisher’s LSD *post hoc* test (**a–k**). * *P* < 0.05, ** *P* < 0.01, *** *P* < 0.001, **** *P* < 0.0001 vs. LFD. # *P* < 0.05 vs. HFD group.

Serine and glycine restriction is widely used to modulate health outcomes in mice and is relatively well-tolerated for several months^16,19–22^. To model the effect of systemic serine deficiency in the context of diet-induced obesity, we fed mice low-fat (10% kcal) (LFD) or high-fat (60% kcal from fat) (HFD) diets and compared their phenotypes to mice fed isonitrogenous diets lacking serine and glycine (−SG LFD or −SG HFD) (Supplementary Table 1). Both −SG diets effectively reduced circulating and hepatic serine and glycine contents during the fed state but not during the fasted state (Fig. 2a,b and Extended Data Fig. 3a,b). The HFD alone reduced liver glycine levels, but to a lesser extent than dietary serine and glycine withdrawal (Extended Data Fig. 3b). Other serine and glycine-derived hepatic metabolites, including glutathione, were not strongly affected by their restriction (Extended Data Fig. 3b). Of note, weight gain caused by HFD was attenuated by dietary serine and glycine restriction, whereas food, calorie and water intake, calorie absorption, and physical activity were all unaffected (Fig. 2c and Extended Data Fig. 3c–h). Using echo magnetic resonance imaging (echoMRI), we quantified lean and fat masses across all groups and observed that dietary serine restriction significantly reduced fat mass but had no effect on lean mass relative to the HFD group (Fig. 2d). Consistent with these changes in adiposity, we observed that HFD feeding increased, whereas serine and glycine restriction decreased, epididymal white adipocyte size (Extended Data Fig. 3i).

To determine how feeding with −SG HFD influences glucose homeostasis, we analysed mice using standard GTTs and ITTs. Whereas −SG HFD attenuated obesity, the mice remained glucose and insulin intolerant (Fig. 2e,f and Extended Data Fig. 3j,k), suggesting that serine and glycine restriction and the consequent reduction in adiposity do not prevent HFD-induced glucose intolerance and insulin resistance. To test whether altered systemic substrate utilization of carbohydrates in preference to lipids were driving the changes in adiposity, we placed mice that had consumed each diet for 18 weeks in metabolic cages and quantified the respiratory exchange ratio (RER). Whereas the HFD altered RER as expected, no changes were observed with dietary serine/glycine restriction (Extended Data Fig. 3l).

**Extended Data Fig. 4:**
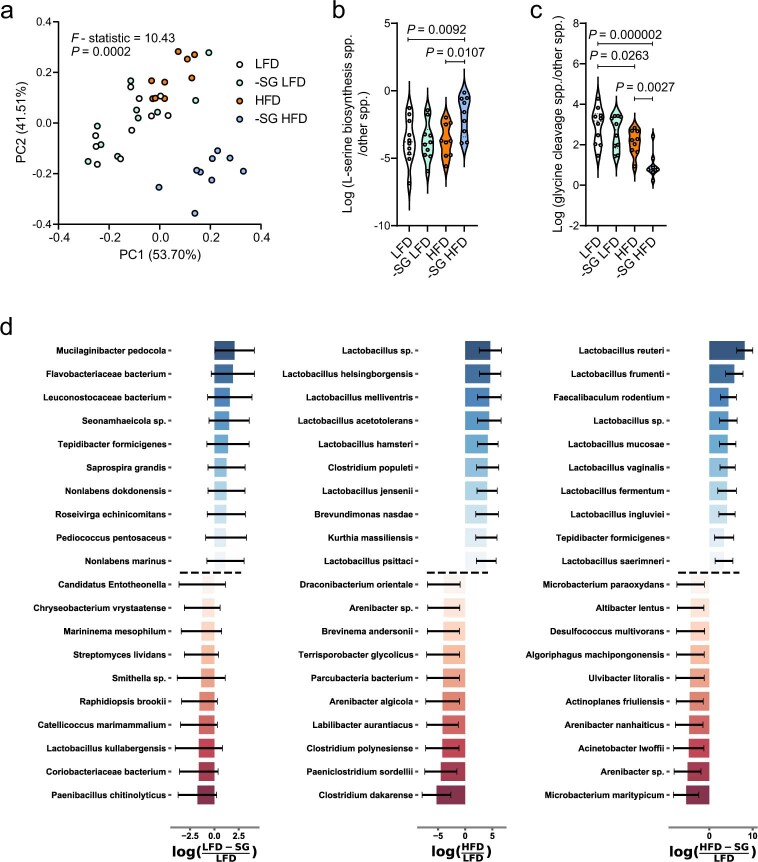
The impact of dietary serine restriction on microbiome composition. (*a*) Robust principal-component analysis of microbiome beta-diversity 18 weeks after feeding mice with a low fat diet (LFD, n = 10), serine/glycine-free LFD (-SG LFD, n = 10), high fat diet (HFD, n = 9), and serine/glycine-free HFD (-SG HFD, n = 9). (*b*) Log-ratio of species with complete vs. incomplete pathways for L-serine biosynthesis 18 weeks after feeding mice with a low fat diet (LFD, n = 10), serine/glycine-free LFD (-SG LFD, n = 10), high fat diet (HFD, n = 9), and serine/glycine-free HFD (-SG HFD, n = 9). (*c*) Log-ratio of species with complete vs. incomplete pathways for glycine cleavage 18 weeks after feeding mice with a low fat diet (LFD, n = 10), serine/glycine-free LFD (-SG LFD, n = 10), high fat diet (HFD, n = 9), and serine/glycine-free HFD (-SG HFD, n = 9). (*d*) Log fold-change of microbiome species 18 weeks after feeding mice with a low fat diet (LFD, n = 10), serine/glycine-free LFD (-SG LFD, n = 10), high fat diet (HFD, n = 9), and serine/glycine-free HFD (-SG HFD, n = 9). Data are presented as a minimum/maximum (**b–c**) and were analyzed using a PERMANOVA test (*a*) and a two-way ANOVA with Fisher’s LSD *post hoc* test (**b–c**).

Next, we performed a metagenomic analysis of faecal microbiota to understand how the above diets affected microbiome composition and diversity, which correlate with and can buffer against dietary deficiencies^23^. Sustained HFD or −SG HFD feeding reduced phylogenetic alpha diversity relative to LFD-fed mice, whereas −SG LFD feeding increased alpha diversity (Fig. 2g). Conversely, PERMANOVA testing of Aitchison distances from robust principal component analysis revealed significant beta-diversity differences between −SG HFD-fed mice and other groups, highlighting the distinct effect of this low-carbohydrate, high-fat, serine and glycine-restricted diet on the faecal microbiome (Extended Data Fig. 4a). Furthermore, the log ratios of strains of microorganisms expressing complete serine biosynthesis and glycine cleavage pathways were increased and decreased, respectively, by −SG HFD (Extended Data Fig. 4b,c), and this diet reduced the log ratio of strains expressing a complete fatty acid synthesis pathway (Fig. 2h). Key strains showing the strongest alterations are listed in Extended Data Fig. 4d. Notably, −SG LFD feeding did not modulate strains in this manner, presumably owing to the higher carbohydrate content, which could facilitate serine synthesis.

**Extended Data Fig. 5:**
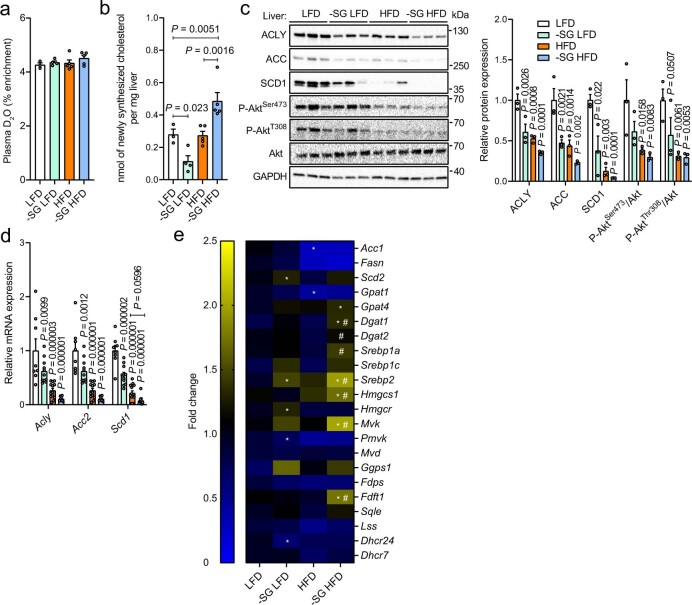
Dietary serine restriction remodels fatty acid and cholesterol synthesis. (*a*) Plasma heavy water (D_2_O) enrichment in mice fed a with low fat diet (LFD, n = 3), serine/glycine-free LFD (-SG LFD, n = 4), high fat diet (HFD, n = 5), and serine/glycine-free HFD (-SG HFD, n = 5). (*b*) *De novo* cholesterol synthesis in mice fed with LFD (n = 3), -SG LFD (n = 4), HFD (n = 5), and -SG HFD (n = 5) for 18 weeks. (*c*) Hepatic protein expression of ATP-citrate lyase (ACLY), acetyl-CoA carboxylase (ACC), and steroyl-CoA desaturase 1 (SCD1), P-Akt^S473^, and P-Akt^T308^ (n = 3 per group). (*d*) Hepatic mRNA expression of ATP-citrate lyase (*Acly*), acetyl-CoA carboxylase (*Acc2*), and steroyl-CoA desaturase 1 (*Scd1*) in mice fed with a low fat diet (LFD, n = 8), serine/glycine-free LFD (-SG LFD, n = 11), high fat diet (HFD, n = 13), and serine/glycine-free HFD (-SG HFD, n = 10). (*e*) Hepatic mRNA expression of enzymes involved in fatty acid and cholesterol synthesis in mice fed with a low fat diet (LFD, n = 8), serine/glycine-free LFD (-SG LFD, n = 11), high fat diet (HFD, n = 13), and serine/glycine-free HFD (-SG HFD, n = 10). Data are mean ± standard error of mean (SEM) and were analyzed using a two-way ANOVA with Fisher’s LSD *post hoc* test (**a–e**). * *P* < 0.05, ** *P* < 0.01, *** *P* < 0.001, **** *P* < 0.0001 vs. LFD. # *P* < 0.05 vs. HFD group.

Next, to directly investigate how serine deficiency affects hepatic lipogenesis, mice fed the above diets for 18 weeks were administered heavy water (D_2_O) for 18 h, and lipids were extracted for quantification of isotope enrichment, molar abundance, and synthesis^24^. Dietary serine and glycine restriction potently reduced hepatic palmitate synthesis by around 70% relative to serine-replete control diets (Fig. 2i and Extended Data Fig. 5a). Hepatic cholesterol synthesis was increased in −SG HFD compared with HFD (Extended Data Fig. 5b). Consistent with these changes, hepatic expression of ATP-citrate lyase (ACLY), acetyl-CoA carboxylase (ACC2) and stearoyl-CoA desaturase (SCD1) were strongly reduced (around 50%) by dietary serine restriction, whereas the expression of cholesterol biosynthesis enzymes was unchanged or increased (Extended Data Fig. 5c–e). Changes in AKT phosphorylation at Ser473 and Thr308 correlated with dietary fat and carbohydrate content rather than serine and glycine levels, further suggesting that serine restriction drives changes in fatty acid metabolism that are independent from insulin signalling (Extended Data Fig. 5c).

## Serine restriction and HFD accelerate neuropathy

**Extended Data Fig. 6:**
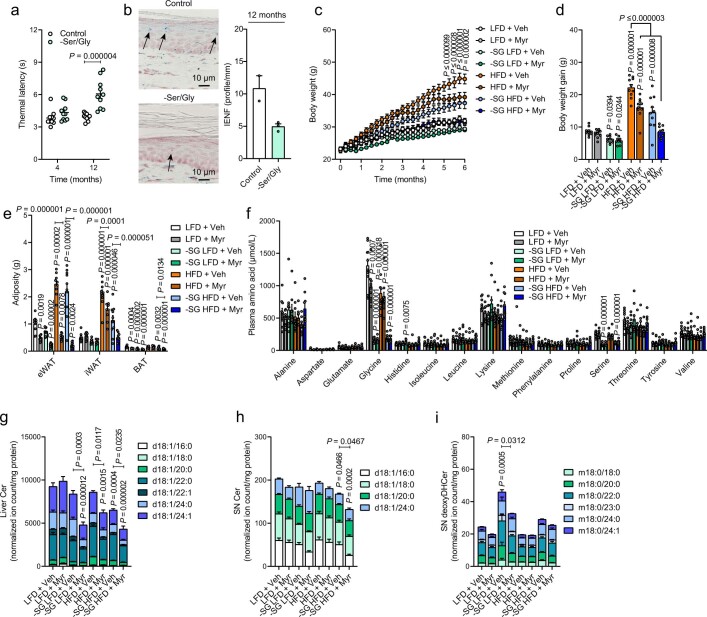
Myriocin remodels obesity and sphingolipid metabolism in serine-associated peripheral neuropathy. (*a*) Thermal latency quantification in C57BL/6J mice fed with either a control diet (n = 10) or serine/glycine-free how (-Ser/Gly, n = 10) for 12 months. (*b*) Representative images of intraepidermal nerve fiber (IENF) density and quantification of IENF in paw skin in mice fed with either a control (n = 2) or serine/glycine free diet (n = 3) for 12 months. PGP9.5 staining of IENF is shown by arrows. (*c*) Body weight time course in mice fed with a low fat diet + vehicle (LFD+Veh, n = 10), LFD + 0.3 mg/kg myriocin (LFD+Myr, n = 10), serine/glycine-free LFD + vehicle (-SG LFD+Veh, n = 10), -SG LFD + myriocin (-SG LFD+Myr, n = 9), high fat diet + vehicle (HFD+Veh, n = 10), HFD + myriocin (HFD+Myr, n = 10), serine/glycine-free HFD + vehicle (-SG HFD+Veh, n = 10), and -SG HFD + myriocin (-SG HFD+Myr, n = 9). (*d*) Body weight gain 6 months after dietary intervention in mice fed with a low fat diet + vehicle (LFD+Veh, n = 10), LFD + 0.3 mg/kg myriocin (LFD+Myr, n = 10), serine/glycine-free LFD + vehicle (-SG LFD+Veh, n = 10), -SG LFD + myriocin (-SG LFD+Myr, n = 9), high fat diet + vehicle (HFD+Veh, n = 10), HFD + myriocin (HFD+Myr, n = 10), serine/glycine-free HFD + vehicle (-SG HFD+Veh, n = 10), and -SG HFD + myriocin (-SG HFD+Myr, n = 9). (*e*) Adipose tissue size 6 months after dietary intervention in mice fed with a low fat diet + vehicle (LFD+Veh, n = 9), LFD + 0.3 mg/kg myriocin (LFD+Myr, n = 10), serine/glycine-free LFD + vehicle (-SG LFD+Veh, n = 10), -SG LFD + myriocin (-SG LFD+Myr, n = 9), high fat diet + vehicle (HFD+Veh, n = 10), HFD + myriocin (HFD+Myr, n = 10), serine/glycine-free HFD + vehicle (-SG HFD+Veh, n = 10), and -SG HFD + myriocin (-SG HFD+Myr, n = 9). (*f*) Plasma amino acid concentration 6 months after dietary intervention in mice fed a with low fat diet + vehicle (LFD+Veh, n = 10), LFD + 0.3 mg/kg myriocin (LFD+Myr, n = 10), serine/glycine-free LFD + vehicle (-SG LFD+Veh, n = 10), -SG LFD + myriocin (-SG LFD+Myr, n = 9), high fat diet + vehicle (HFD+Veh, n = 10), HFD + myriocin (HFD+Myr, n = 10), serine/glycine-free HFD + vehicle (-SG HFD+Veh, n = 10), and -SG HFD + myriocin (-SG HFD+Myr, n = 9). (*g*) Stack plot of liver ceramides in mice fed with a low fat diet + vehicle (LFD+Veh, n = 10), LFD + 0.3 mg/kg myriocin (LFD+Myr, n = 10), serine/glycine-free LFD + vehicle (-SG LFD+Veh, n = 10), -SG LFD + myriocin (-SG LFD+Myr, n = 9), high fat diet + vehicle (HFD+Veh, n = 10), HFD + myriocin (HFD+Myr, n = 10), serine/glycine-free HFD + vehicle (-SG HFD+Veh, n = 10), and -SG HFD + myriocin (-SG HFD+Myr, n = 9). (*h*) Stack plot of sciatic nerve ceramides in mice fed with a low fat diet + vehicle (LFD+Veh, n = 10), LFD + 0.3 mg/kg myriocin (LFD+Myr, n = 10), serine/glycine-free LFD + vehicle (-SG LFD+Veh, n = 10), -SG LFD + myriocin (-SG LFD+Myr, n = 9), high fat diet + vehicle (HFD+Veh, n = 10), HFD + myriocin (HFD+Myr, n = 10), serine/glycine-free HFD + vehicle (-SG HFD+Veh, n = 10), and -SG HFD + myriocin (-SG HFD+Myr, n = 9). (*i*) Stack plot of sciatic nerve deoxydihydroceramide (deoxyDHCer) in mice fed a with low fat diet + vehicle (LFD+Veh, n = 10), LFD + 0.3 mg/kg myriocin (LFD+Myr, n = 10), serine/glycine-free LFD + vehicle (-SG LFD+Veh, n = 10), -SG LFD + myriocin (-SG LFD+Myr, n = 9), high fat diet + vehicle (HFD+Veh, n = 10), HFD + myriocin (HFD+Myr, n = 10), serine/glycine-free HFD + vehicle (-SG HFD+Veh, n = 10), and -SG HFD + myriocin (-SG HFD+Myr, n = 9). Data are mean ± standard error of mean (SEM) and were analyzed using a two-way ANOVA with Fisher’s LSD *post hoc* test (**a** and **c**) and a one-way ANOVA with Fisher’s LSD *post hoc* test (**d–i**). Statistical analyses in g-i were performed using summed sphingolipid abundances.

Systemic serine deficiency has recently been linked to various neurodegenerative disorders^16,25–28^. People with diabetes who have elevated serine disposal could therefore be more susceptible to neurological co-morbidities reminiscent of serine-associated peripheral neuropathy. To determine whether long-term, chronic serine deficiency is sufficient to drive peripheral neuropathy, we fed mice either control or serine- and glycine-free chow diets (19.2% of energy from fat) for up to 12 months. Temporal quantification of the thermal response to heat revealed progression to hypoalgesia after 12 months of dietary intervention (Extended Data Fig. 6a), consistent with previous observations^16^. At this time point we also detected reduced intraepidermal nerve fibre (IENF) density in paw skin (Extended Data Fig. 6b), which is also a clinical measure of small sensory fibre degeneration^29^. Of note, mice fed −SG HFD for just three months exhibited marked thermal hypoalgesia (Fig. 2j), indicating that a combination of low systemic serine and HFD accelerate the onset of peripheral neuropathy in mice.

**Fig. 3:**
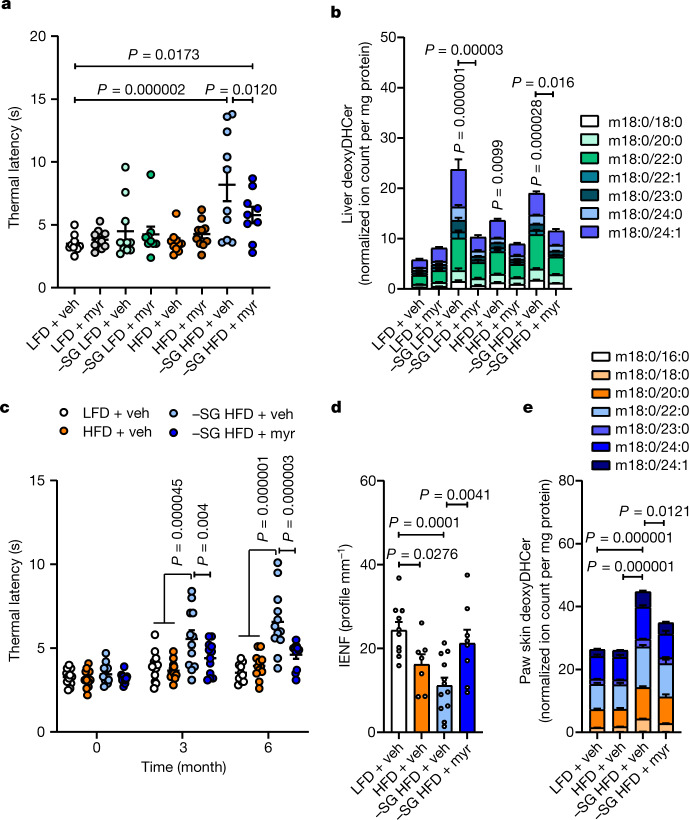
Inhibition of de novo sphingolipid biosynthesis decelerates the kinetics of serine-associated peripheral neuropathy. **a**, Thermal latency in mice fed with LFD plus vehicle (veh) (*n* = 10), LFD plus 0.3 mg kg^−1^ myriocin (myr) (*n* = 10), −SG LFD plus vehicle (*n* = 10), −SG LFD plus myriocin (*n* = 9), HFD plus vehicle (*n* = 10), HFD plus myriocin (*n* = 10), −SG HFD plus vehicle (*n* = 10) or −SG HFD plus myriocin (*n* = 9). **b**, Stack plot of liver deoxyDHCer in mice fed with LFD plus vehicle (*n* = 10), LFD plus myriocin (*n* = 10), −SG LFD plus vehicle (*n* = 10), −SG LFD plus myriocin (*n* = 9), HFD plus vehicle (*n* = 10), HFD plus myriocin (*n* = 10), −SG HFD plus vehicle (*n* = 10) or −SG HFD plus myriocin (*n* = 9). **c**, Thermal latency time course in mice fed with LFD plus vehicle (*n* = 12), HFD plus vehicle (*n* = 12), −SG HFD plus vehicle (*n* = 12) or −SG HFD plus myriocin (*n* = 11). **d**, IENF density in mice fed with LFD plus vehicle (*n* = 10), HFD plus vehicle (*n* = 7), −SG HFD plus vehicle (*n* = 12) or −SG HFD plus myriocin (*n* = 8). **e**, Paw skin deoxyDHCer distribution in mice fed with LFD plus vehicle (*n* = 12), HFD plus vehicle (*n* = 12), −SG HFD plus vehicle (*n* = 12) or −SG HFD plus myriocin (*n* = 11). Data are mean ± s.e.m., and were analysed using one-way ANOVA with Fisher’s least significant difference post hoc test (**a**,**b**,**d**,**e**) or two-way ANOVA with Fisher’s least significant difference post hoc test (**c**). Statistical analyses in **b**,**e** were performed using summed deoxyDHCer abundances.

Serine is essential for the biosynthesis of canonical sphingolipids, which are enriched in the nervous system. When serine becomes limited, serine palmitoyltransferase (SPT) incorporates other amino acids, including alanine, to form non-canonical deoxysphingolipids^30,31^. Given the importance of canonical ceramides and 1-deoxy(dihydro)ceramides in obesity and neuropathy, respectively^15,32^, we hypothesized that SPT inhibition could influence the observed obesity and neuropathy phenotypes. We therefore administered myriocin (0.3 mg kg^−1^ every other day), an inhibitor of SPT, or vehicle to mice fed the above diets for 6 months and quantified sphingolipid diversity and thermal sensing. Consistent with previous reports^32^, myriocin treatment attenuated HFD-induced weight gain without affecting plasma serine and glycine levels (Extended Data Fig. 6c–f) However, myriocin also mitigated thermal hypoalgesia exhibited by mice fed −SG HFD (Fig. 3a), suggesting that a reduction in SPT activity reduced serine-associated peripheral neuropathy.

**Extended Data Fig. 7:**
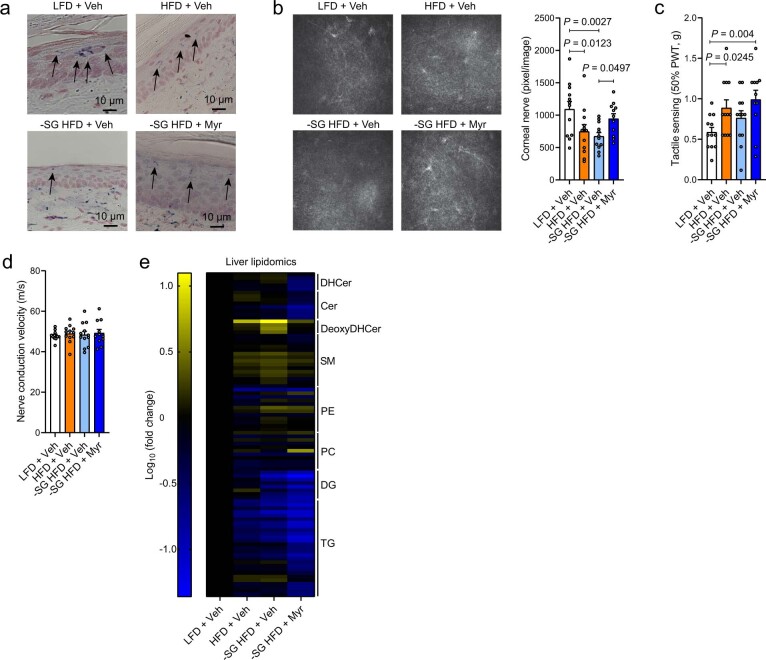
Myriocin improves innervation in the context of serine deficiency and alters the hepatic lipidome. (*a*) Representative images of intraepidermal nerve fiber (IENF) density in mice fed a with low fat diet + vehicle (LFD+Veh, n = 10), high fat diet + vehicle (HFD+Veh, n = 7), serine/glycine free HFD + vehicle (-SG HFD+Veh, n = 12), and -SG HFD + myriocin (-SG HFD+Myr, n = 8). (*b*) Representative images and quantification of corneal nerves in mice fed a with low fat diet + vehicle (LFD+Veh, n = 12), high fat diet + vehicle (HFD+Veh, n = 12), serine/glycine-free HFD + vehicle (-SG HFD+Veh, n = 12), and -SG HFD + myriocin (-SG HFD+Myr, n = 11). (*c*) Tactile sensing in mice fed with a low fat diet + vehicle (LFD+Veh, n = 12), high fat diet + vehicle (HFD+Veh, n = 12), serine/glycine-free HFD + vehicle (-SG HFD+Veh, n = 12), and -SG HFD + myriocin (-SG HFD+Myr, n = 11). (*d*) Motor nerve conduction velocity in mice fed with a low fat diet + vehicle (LFD+Veh, n = 12), high fat diet + vehicle (HFD+Veh, n = 12), serine/glycine-free HFD + vehicle (-SG HFD+Veh, n = 12), and -SG HFD + myriocin (-SG HFD+Myr, n = 11). (*e*) Liver high-resolution lipidomics analysis in the fed state in mice fed with a low fat diet + vehicle (LFD+Veh, n = 12), high fat diet + vehicle (HFD+Veh, n = 12), serine/glycine-free HFD + vehicle (-SG HFD+Veh, n = 12), and -SG HFD + myriocin (-SG HFD+Myr, n = 11). Data are mean ± standard error of mean (SEM) and were analyzed using a one-way ANOVA with Fisher’s LSD *post hoc* test (**b–e**).

To better understand the metabolic drivers of this peripheral neuropathy phenotype, we next quantified ceramides and deoxydihydroceramides (deoxyDHCer) in liver and sciatic nerve. Restriction of serine and glycine increased deoxyDHCer in LFD and HFD settings and reduced canonical ceramides in HFD background, whereas myriocin generally reduced abundances of sphingolipids (Fig. 3b and Extended Data Fig. 6g–i). By contrast, ceramide and 1-deoxysphingolipid levels were unaltered or did not correlate with peripheral neuropathy phenotype in sciatic nerve, potentially owing to the large lipid deposits present in myelin (Extended Data Fig. 6h,i). Notably, serine restriction in a LFD background did not induce thermal hypoalgesia after six months (Fig. 3a), indicating that 1-deoxysphingolipid accumulation alone is insufficient to drive this phenotype and consistent with recent reports of an *Sptlc1*^*C133W*^ mouse model^33^. Next, we measured IENF density, corneal nerve density, and tissue lipids in a separate cohort of mice fed LFD, HFD, −SG HFD or −SG HFD plus myriocin for six months. Myriocin mitigated the onset of thermal hypoalgesia in mice fed −SG HFD (Fig. 3c), and this treatment also protected small fibre nerve density in the epidermis and cornea (Fig. 3d and Extended Data Fig. 7a,b) without affecting tactile sensing or MNCV (Extended Data Fig. 7c,d), suggesting this early-onset phenotype is specific to small sensory fibres and 1-deoxysphingolipids, in contrast to other nodes in sphingolipid metabolism^34^. Mice fed −SG HFD exhibited increased hepatic 1-deoxysphingolipids and sphingomyelin, whereas myriocin strongly reduced the levels of sphingolipids as well as triglycerides and diacylglycerides (Extended Data Fig. 7e), further highlighting the pleiotropic effects of this molecule on the lipidome^32,35^. Finally, paw skin exhibited a significant increase in deoxyDHCer that was reduced with myriocin treatment (Fig. 3e), suggesting the lipid microenvironment surrounding small fibres can influence sensory function.

**Extended Data Fig. 8:**
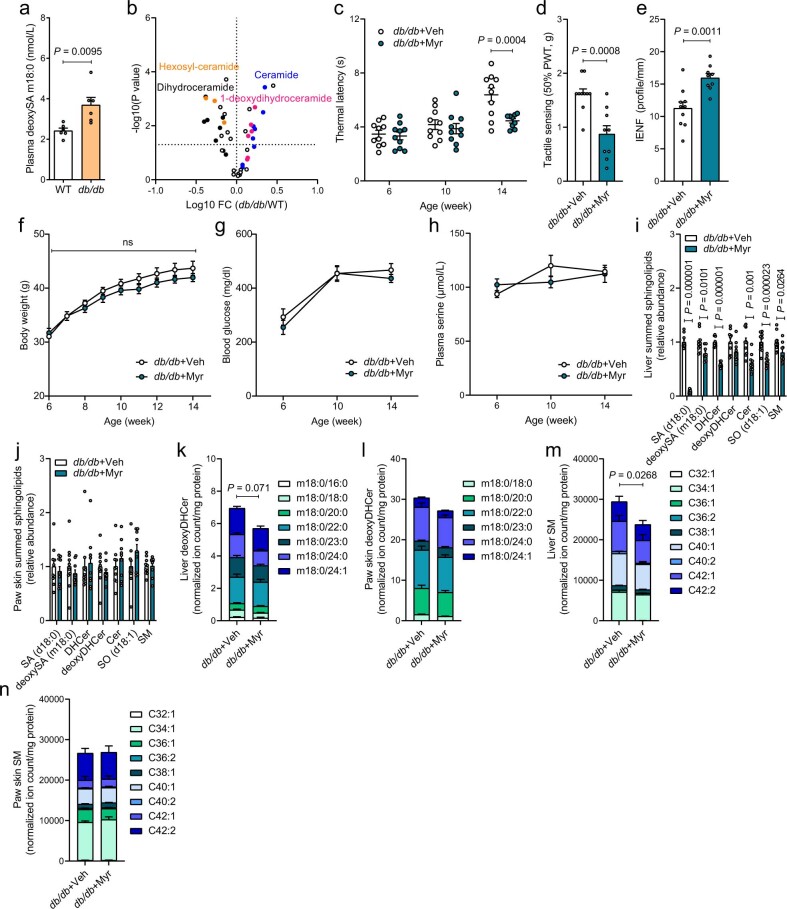
Myriocin slows the progression of peripheral neuropathy in BKS-*db/db* mice. **a**) Hydrolyzed plasma deoxysphinganine (deoxySA) concentration in 16-week-old WT and BKS-*db/db* mice (n = 6 per group). **b**) Hepatic targeted sphingolipid determination 16-week-old WT and BKS-*db/db* mice (n = 6 per group). **c**) Thermal latency time-course in BKS-*db/db* mice treated with either vehicle (n = 10) or myriocin (0.3 mg/kg every other day, n = 9) for 8 weeks. **d**) Tactile sensing in BKS-*db/db* mice treated with either vehicle (n=10) or myriocin (n = 9) for 8 weeks. **e**) Intraepidermal nerve fiber density in BKS-*db/db* mice treated with either vehicle (n = 10) or myriocin (n = 9) for 8 weeks. **f**) Body weight time-course in BKS-*db/db* mice treated with either vehicle (n = 10) or myriocin (n = 9) for 8 weeks. **g**) Fasting (6-h) blood glucose time-course in BKS-*db/db* mice treated with either vehicle (n = 10) or myriocin (n = 9) for 8 weeks. **h**) Fasting (6-h) plasma serine time-course in BKS-*db/db* mice treated with either vehicle (n = 10) or myriocin (n = 8) for 8 weeks. **i**) Summed hepatic sphingolipids in BKS-*db/db* mice treated with either vehicle (n = 10) or myriocin (n = 9) for 8 weeks. **j**) Summed paw skin sphingolipids in BKS-*db/db* mice treated with either vehicle (n = 10) or myriocin (n = 9) for 8 weeks. **k**) Stack plot of liver deoxydihydroceramide (deoxyDHCer) species in BKS-*db/db* mice treated with either vehicle (n = 10) or myriocin (n = 9) for 8 weeks. **l**) Stack plot of paw skin deoxydihydroceramide (deoxyDHCer) species in BKS-*db/db* mice treated with either vehicle (n = 10) or myriocin (n = 9) for 8 weeks. m) Stack plot of liver sphingomyelin (SM) species in BKS-*db/db* mice treated with either vehicle (n = 10) or myriocin (n = 9) for 8 weeks. **n**) Stack plot of paw skin sphingomyelin (SM) species in BKS-*db/db* mice treated with either vehicle (n = 10) or myriocin (n = 9) for 8 weeks. Data are mean ± standard error of mean (SEM) and were analyzed using a two-sided independent t-test (**a–b**,**d–e**, **i–n**) and a two-way ANOVA with Fisher’s LSD *post hoc* test (**c,f–h**). Statistical analyses in k-n were performed using summed sphingolipid abundances.

We next evaluated whether suppressing SPT activity could mitigate neuropathy in *db/db* mice, which have increased circulating deoxySA as well as increased hepatic ceramides and deoxyDHCer (Extended Data Fig. 8a,b). Consistent with results in the −SG HFD diet-induced peripheral neuropathy model, administration of myriocin at six weeks of age prevented progression to thermal hypoalgesia and restored tactile sensation in *db/db* mice (Extended Data Fig. 8c,d). IENF density was also increased in *db/db* mice dosed for eight weeks with myriocin (Extended Data Fig. 8e). Although myriocin did not affect body weight gain, hyperglycaemia or plasma serine levels (Extended Data Fig. 8f–h), it strongly reduced canonical sphingolipids in the liver but showed limited effects on paw skin 1-deoxysphingolipids and ceramides (Extended Data Fig. 8i–n). Thus, myriocin probably acts through both direct and indirect mechanisms targeting liver and other tissues, which also accounts for its toxicity^35^.

## Serine supplementation slows neuropathy

**Fig. 4:**
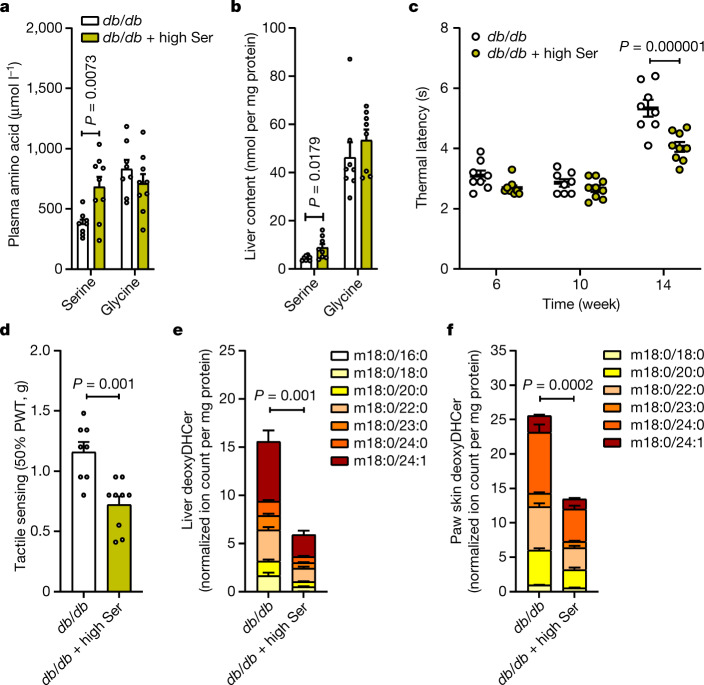
Dietary serine supplementation reduces deoxysphingolipid content and slows down peripheral neuropathy. **a**, Plasma amino acid levels in the fed state in BKS-*db/db* mice fed with either a control (*n* = 8) or serine-supplemented diet (*n* = 9) for 8 weeks. **b**, Liver amino acid content in the fed state in BKS-*db/db* mice fed with either a control or serine-supplemented diet for 8 weeks (*n* = 8 per group). **c**, Thermal latency time course in BKS-*db/db* mice fed with either a control (*n* = 8) or serine-supplemented diet (*n* = 9). **d**, Tactile sensing 8 weeks after feeding BKS-*db/db* mice with either a control (*n* = 8) or serine-supplemented diet (*n* = 9). **e**, Levels of deoxyDHCer in the liver in BKS-*db/db* mice fed with either a control or serine-supplemented diet for 8 weeks (*n* = 8 per group). **f**, Levels of deoxyDHCer in the paw skin of BKS-*db/db* mice fed with either a control or serine-supplemented diet for 8 weeks (*n* = 8 per group). Data are mean ± s.e.m., and were analysed using a two-sided independent *t*-test (**a**,**b**,**d**–**f**) or two-way ANOVA with Fisher’s least significant difference post hoc test (**c**). Statistical analyses in **e**,**f** were performed using summed deoxyDHCer abundances.

**Extended Data Fig. 9:**
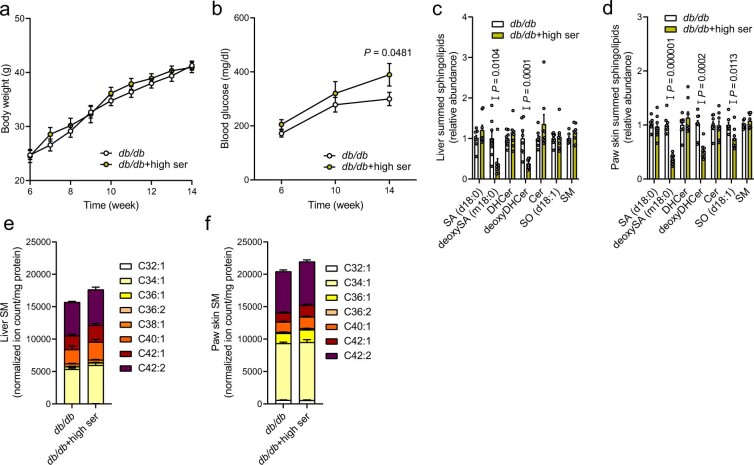
Dietary serine supplementation mitigates 1-deoxysphingolipid in BKS-*db/db* liver and skin. **a**) Body weight time-course in BKS-*db/db* mice fed with either a control (n = 8) or serine supplemented diet (n = 9) for 8 weeks. **b**) Fasting (6-h) blood glucose time-course in BKS-*db/db* fed with either a control (n = 8) or serine supplemented diet (n = 9) for 8 weeks. **c**) Summed hepatic sphingolipids in BKS-*db/db* mice fed with either a control or serine supplemented diet for 8 weeks (n = 8 per group). **d**) Summed paw skin sphingolipids in BKS-*db/db* mice fed with either a control or serine supplemented diet for 8 weeks (n = 8 per group). **e**) Levels of sphingomyelin species (SM) in the liver in BKS-*db/db* mice fed with either a control or serine supplemented diet for 8 weeks (n = 8 per group). **f**) Levels of sphingomyelin species (SM) in the paw skin in BKS-*db/db* mice fed with either a control or serine supplemented diet for 8 weeks (n = 8 per group). Data are mean ± standard error of mean (SEM) and were analyzed with a two-way ANOVA with Fisher’s LSD *post hoc* test (**a–b**) and a two-sided independent t-test (**c–f**). Statistical analyses in e-f were performed using summed abundances.

Considering the chronic serine deficiency exhibited by diabetic mice, we next fed *db/db* mice a 3% serine-enriched diet starting at 6 weeks of age and quantified neuropathy phenotypes. Thermal sensation was measured at 6, 10 and 14 weeks of age and tactile sensation at time of sacrifice, at which point mice exhibited elevated plasma and hepatic serine, but not glycine, levels (Fig. 4a,b). We observed no change in body weight and a slight increase in circulating glucose levels in this cohort (Extended Data Fig. 9a,b). However, both thermal and tactile hypoalgesia were reduced in mice fed this serine-enriched diet (Fig. 4c,d). Of note, no change in canonical sphingolipids was observed across tissues, yet 1-deoxysphingolipids were robustly decreased in both liver and paw skin (Fig. 4e,f and Extended Data Fig. 9c–f). Collectively, these data suggest that supplementation of serine can slow the progression of diabetic peripheral neuropathy.

## Discussion

Here we describe how direct or indirect induction of chronic, systemic serine deficiency alters lipid homeostasis and contributes to diabetic peripheral neuropathy. Modulating dyslipidaemia with myriocin or 1-deoxysphingolipid biosynthesis with serine supplementation both mitigate thermal and tactile hypoalgesia in obese diabetic mice. These results highlight how serine deficiency can synergize with dyslipidaemia to alter neurological phenotypes both in rare disease contexts^16,25,26^ and indirectly via a widespread, chronic disease such as type 2 diabetes, where it manifests as a co-morbidity experienced by a subset of patients. Reduced circulating serine and glycine in diabetic mice may be driven by increased flux through gluconeogenesis, one-carbon metabolism, renal retention^18,36^ and/or disposal as acylglycines^37^, which are also influenced by dyslipidaemia^38^.

A STT, analogous to an oral glucose tolerance test (OGTT), could identify patients that exhibit elevated, postprandial serine disposal and who might be particularly susceptible to sensory neuropathy. Normalizing circulating serine levels via dietary supplementation delays the onset and progression of sensory neuropathy in *db/db* mice. Indeed, supplementation of serine and B vitamins improves peripheral neuropathy in some pre-clinical models and are the focus of clinical trials for various neurodegenerative disorders^39–41^ (ClinicalTrials.gov: NCT03062449). Our results highlight physiologically relevant molecular links between serine and glycine homeostasis, sphingolipid metabolism, and diabetic co-morbidities. The metabolic and neuropathic phenotypes of mouse models of diabetes and obesity vary across strains and genotypes^42,43^, and C57BL6/J mice are particularly sensitive to the metabolic consequences of HFD owing to mutations in *Nnt*^44^ and other genes. However, our data in both diabetic *db/db* mice and in C57BL/6J mice fed −SG HFD demonstrate that serine deficiency combined with dyslipidaemia can drive peripheral neuropathy in different genetic backgrounds.

Several key questions remain, including why serine and glycine deprivation suppresses hepatic fatty acid synthesis and gene expression in the liver. In addition, diverse sphingolipid species and/or their mis-localization contribute to neuropathy^21,45–48^, but their exact mechanisms of toxicity remain unclear. We have developed and validated a dietary model of serine-associated sensory neuropathy that develops a phenotype in three months, which could aid in understanding how neurotoxic dyslipidaemia can be managed. Diverse genetic changes may influence circulating serine and glycine, including common single nucleotide polymorphisms and rare coding events^25,28,49^, or such deficiencies could be induced by diabetes-induced rewired hepatic metabolism. Thus, in both altering sphingolipid diversity and compromising the liver’s ability to handle nutritional lipid overload, systemic serine deficiency emerges as a modifier of age- and diabetes-associated neuropathies.

## Methods

### Mouse experiments

All mouse experiments were approved and conducted in accordance with the Institutional Animal Care and Use Committee (IACUC) of the University of California, San Diego and the Salk Institute for Biological Studies. Mice were housed in the same room ensuring exposure to the same temperature (21 °C), humidity (ambient humidity 65%) and a 12-h light:dark cycle (06:00–18:00). In Fig. 1, 14- to 16-week-old C57BL/6J (JAX 000664) or BKS-*db/db* mice (JAX 000642), and 10- to 12-week-old vehicle- or STZ-treated C57BL/6J (JAX 000664) mice were fasted for 6 h prior to tissue collection. Animals were anaesthetized with isoflurane, decapitated, and tissues were rapidly collected using Wollenberger clamps pre-cooled to the temperature of liquid nitrogen and stored at −80 °C until analysis. For Fig. 2, 8-week-old C57BL/6J (JAX 000664) were fed with diets obtained from Envigo. Dietary composition is detailed in Supplementary Table 1. In dietary experiments, tissues were collected between 07:00–10:00 h—that is, in the fed state unless stated otherwise. From a separate cohort of animals, plasma samples were collected 18 weeks after dietary intervention in the fed (07:00–10:00 h) and fasted (18-h overnight fast) state. In *db/db* mice experiments, tissues were collected after 6-h fasting. For Fig. 3, 8-week-old C57BL/6J (JAX 000664) were fed with diets obtained from Envigo. Tissue collection took place between 07:00–10:00 h. Animals were anaesthetized with isoflurane, decapitated, and tissues were rapidly collected using Wollenberger clamps pre-cooled to the temperature of liquid nitrogen and stored at −80 °C until analysis. For Fig. 4, 6-week-old BKS-*db/db* mice (JAX 000642) were fed with either a control or serine-supplemented diet (provided by Envigo) for a period of 8 weeks. Tissue collection took place between 07:00–10:00 h unless stated otherwise. Animals were anaesthetized with isoflurane, decapitated, and tissues rapidly collected using Wollenberger clamps pre-cooled to the temperature of liquid nitrogen and stored at −80 °C until analysis.

### Serine tolerance test

Age-matched 14- to 16-week-old wild-type and BKS-*db/db*, and 10- to 12-week-old vehicle- and STZ-treated C57BL/6J (JAX 000664) mice were fasted overnight with water access provided ad libitum. For a STT, animals were weighed, and serine and/or glucose were administered via oral gavage at a dose of 400 mg kg^−1^ and 2 g kg^−1^, respectively, with tail tip blood samples collected into EDTA-coated microvette tubes (Sarstedt) before, and 15, 30, 60, 120 and 180 min after an oral gavage. EDTA microvettes were spun at 2,000*g* at 4 °C for 5 min to obtain plasma, and samples stored at −80 °C until analysis. Blood glucose and serine concentrations were quantified using Contour Next glucometer (Bayer) and gas chromatography–mass spectrometry as described below, respectively. Plasma serine pharmacokinetics were determined for a 400 mg kg^−1^ dose using PK solver^50^.

To qualify downstream fate of serine, wild-type mice were fasted overnight, weighed in the morning, and [U-^13^C_3_]serine administered via oral gavage at a dose of 400 mg kg^−1^, with tissues collected, using Wollenberger clamps pre-cooled to the temperature of liquid nitrogen, before, and 15, 30, 45, 60, and 120 min after oral gavage, and samples stored at −80 °C until analysis.

### Serum insulin and glucagon measurements

Commercially available kits were used to determine serum insulin (Mouse Insulin ELISA 10-1247-01, Mercodia) and glucagon (Glucagon ELISA 10-1271-01, Mercodia) following a 6-h fast in mice according to the manufacturer’s instructions.

### Lipogenesis D_2_O experiments

C57BL/6J mice fed diets for 18 weeks were injected intraperitoneally with D_2_O (in 0.9% NaCl) at a dose of 0.027 ml per g of body weight with drinking water replaced with 6% D_2_O-enriched solution for a period of ~18 h. In the morning (07:00–10:00 h) tissues were rapidly collected using Wollenberger clamps pre-cooled to the temperature of liquid nitrogen and stored at −80 °C until analysis.

Plasma D_2_O enrichment was determined using deuterium–acetone exchange protocol as previously described^24^. In brief, 5 µl of plasma were incubated with 4 µl of 5% acetone in acetonitrile solution and 4 µl of 10 M NaOH for 24 h. Next, 500 mg of Na_2_SO_4_ and 600 µl of chloroform were added, and samples vortex-mixed. After 2 min centrifugation at 3,000*g*, 80 µl was transferred in triplicate into gas chromatography–mass spectrometry (GC–MS) vials, and plasma D_2_O enrichment was quantified from an external standard curve on an Agilent DB-35MS column (30 m by 0.25 mm internal diameter × 0.25 μm, Agilent J&W Scientific) installed in an Agilent 7890 A gas chromatograph (GC) interfaced with an Agilent 5975 C mass spectrometer with the following temperature program: 60 °C initial, increase by 20 °C min^−1^ to 100 °C, increase by 50 °C min^−1^ to 220 °C, and hold for 1 min.

To quantify tissue D_2_O labelling, ~20 mg of frozen tissue was homogenized with 250 µl −20 °C methanol, 250 µl ice-cold saline and 500 µl −20 °C chloroform spiked with internal standards palmitate-d_31_ (Cambridge Isotope Laboratories, DLM-215-PK) and coprostanol (Sigma, 7578). After a 5 min spin at 4 °C at 21,000*g*, the chloroform fraction was collected, dried, and resuspended with 500 µl of 2% H_2_SO_4_ in methanol for 2 h at 50 °C. Next, 100 µl of saturated NaCl and 500 µl of hexane were added, samples vortex-mixed, and upper hexane phase collected and transferred into a GC–MS vial. Fatty acid methyl esters were analysed using a Select FAME column (100 m × 0.25 mm internal diameter) installed in an Agilent 7890 A GC interfaced with an Agilent 5975 C MS using the following temperature program: 80 °C initial, increase by 20 °C min^−1^ to 170 °C, increase by 1 °C min^−1^ to 204 °C, then 20 °C min^−1^ to 250 °C and hold for 10 min. The percent isotopologue distribution of each fatty acid and polar metabolite was determined and corrected for natural abundance using in-house algorithms adapted from a previous report^51^.

### GTT and ITT

For GTT and ITT, C57BL/6J mice fed the diets for 18 weeks were fasted overnight with water provided ad libitum. In the morning animals were weighed and fasting blood glucose was determined from a tail bleed using a Contour Next glucometer (Bayer). For GTT, the animals were injected intraperitoneally with a bolus of glucose at a dose of 2 g kg^−1^ of body weight, and blood glucose determined at 15, 30, 60, 120 and 180 min post-injection. For ITT, the animals were injected intraperitoneally with a bolus of insulin (100 IU ml^−1^ Humulin Insulin, Eli Lilly) at a dose of 0.5 IU kg^−1^, and blood glucose was quantified at 15, 30, 60 and 90 min post-injection as previously described^52^.

### Determination of body composition and systemic metabolic rates

Lean and fat masses were determined using a EchoMRI 3-in-1 instrument (quantitative nuclear magnetic resonance (qNMR) imaging system). Comprehensive Laboratory Animal Monitoring System (CLAMS) (Oxymax, Columbus Instruments) was used to quantify systemic metabolic rates in individually housed mice during a period of 6 days. Water, food, and calorie intake were calculated from individually housed animals over a period of 6 days when subjected to CLAMS. Whole-body oxygen consumption (VO_2_) and carbon dioxide (VCO_2_) rates were normalized to corresponding total body weights, and RER was calculated as ratio of VCO_2_ to VO_2_.

### Faecal bomb calorimetry and calorie absorption

Approximately 1 g faeces was desiccated overnight and ground using a mortar and pestle. Powdered sample was reconstituted into a pellet with 300 µl ddH_2_O and weighed. Pellet was placed in bomb cylinder surrounded by 2,000 ml ddH_2_O (Parr 6100 Compensated Jacket Calorimeter). Heat produced by combustion was measured by change in water temperature. The calorimeter energy equivalent, W (Cal °C^−1^), was calculated with standardized benzoic acid. The final energy content of each pellet was calculated as follows:

$${\rm{Gross}}\,{\rm{energy}}=\frac{({T}_{{\rm{final}}}\,\mbox{--}{T}_{{\rm{initial}}})}{{\rm{sample}}\,{\rm{weight}}}$$

Calorie absorption was calculated by subtracting gross energy (faecal calorie extraction) from calorie intake.

### Microbiome analysis

DNA was extracted from 10–30 mg of stool using the MoBio PowerFecal DNA isolation kit (12830-50). Extracted DNA was quantified using a Nanodrop (ThermoFisher Scientific). The whole-genome sequencing raw data was uploaded to Qiita^53^, where we followed their default processing workflow. In summary, the raw reads were adapter filtered using the auto-detect parameters in fastp version 20^54^ and host (mouse) filtered using minimap2 version 2.17^55^. The resulting sequences were aligned using Bowtie 2 version 2.4.2^56^ to the Web of Life (WoL) reference database^57^ via the Web of Life Toolkit App (https://github.com/qiyunzhu/woltka); this step generated tables at genus, species, per genome, and per gene tables. For all analyses we used the per genome table; then for alpha diversity we removed any samples below 1,273,062 sequences per sample and for beta-diversity analysis we rarefied at the same value. Downstream analyses were performed in QIIME 2 version 2020.11^58^. To asses global microbiota alterations, alpha diversity analysis was performed through Faith’s PD^59^ and beta diversity through robust principal component analysis (RPCA)^60^ and resulting Aitchison distances were evaluated through permutational multivariate analysis of variance (PERMANOVA)^61^.

We then designed a Bayesian hierarchical model for differential abundance incorporating diet type as a fixed effect and cage as a random effect. We model the count generating process as a negative binomial distribution to account for overdispersion. Due to the sparsity of microbiome data, we also accounted for zero-inflation by assigning each microbe a probability of being unobserved separately from the count generating process:

$${y}_{ij}=\left\{\begin{array}{cl}0,\quad  & {\theta }_{j}=1\\ {\rm{Negative}}\,{\rm{binomial}}({\eta }_{ij},{\varphi }_{j}),\quad  & {\theta }_{j}=0\end{array}\right.$$

$${\theta }_{j}={\rm{Bernoulli}}({\pi }_{j})$$

$$\log ({\eta }_{ij})={x}_{i}{\beta }_{j}+{z}_{i}{u}_{j}+\,\log ({{\rm{depth}}}_{i})$$

We wrote this model using the Stan probabilistic programming language^62^ and fit the model using BIRDMAn (https://github.com/gibsramen/BIRDMAn). To account for compositionality, we fit this model using the first microbe in the table as an additive log ratio reference and converted log fold changes into centred log ratio coordinates after fitting. We used the following as prior distributions for the target parameters:

$$\begin{array}{c}{\pi }_{j}\sim {\rm{Beta}}(1.5,1.5)\\ {\varphi }_{j}^{-1}\sim {{\rm{Cauchy}}}_{+}(0,3)\\ {\beta }_{j}\sim {\rm{Normal}}(0,5)\\ {u}_{j}\sim {\rm{Normal}}(0,2)\end{array}$$

in which *i* is the sample, *j* is the feature, *y* is the microbial count, *θ* is the indicator for non-biological zero, *η* is the mean feature count, *x* is the covariate, *β* is the regression coefficients to be estimated (log-fold changes), *π* is the probability of non-biological zero, *z* is the cage identifier variable, *u* is the random effect of cage, and *ϕ* is the overdispersion parameter. In order to compare functional changes associated with strain level differential abundances a comparative genomics pathway completeness approach was taken. First, each genome was assessed via MetaCyc^63^ pathway completeness, a proportion ranging from zero to one, by mapping characterized genes to reactions and finally to pathways. Each pathway was then correlated by Spearman’s rank correlation to the beta differential abundance determined from the above model. Serine biosynthesis, glycine cleavage, and fatty acid synthesis pathways were significantly correlated to betas. To validate these correlations, the log ratio of the sum of the abundance of genomes with complete pathways (completeness = 1) vs. those without (completeness < 1) were evaluated between treatment groups.

### Behavioural assays

#### Thermal sensation

Small sensory C fibre function was quantified by behavioural responses to heat using a thermal nociception test device (UARD) as previously described^64^. In brief, the apparatus surface was warmed up to 30 °C, and animals were placed in individual testing chambers for 20–30 min prior to testing. Four separate response latency measurements were performed, and the mean of the last triplicate taken to represent response latency for each animal. All measurements were made on coded animals by an observer unaware of the treatment groups.

#### Tactile sensation

Animals were placed on the von Frey stand and allowed to acclimate for 20–30 min. The range of manual von Frey filaments was used: 2.44, 2.83, 3.22, 3.61, 3.84, 4.08, 4.31, 4.56, 4.74 (Kom Kare). Testing began with the 3.84 filament and the pressure applied was repeated five times. If a positive response was observed, the next lower weighted filament was used in the sequence. In the case of a negative response, the next higher weighted filament was applied. All measurements were made on coded animals by an observer unaware of the treatment groups.

#### Nerve conduction velocity

Conduction of a motor nerve was quantified in anaesthetized mice using EZ Anesthesia Versaflex system (Braintree Scientific, Z-7150). In brief, lightly anaesthetized mice were transferred onto a water-heated pad with anaesthesia maintained via a face mask. Two recording platinum electrodes were inserted between the animal’s second, third, and fourth toes, and a grounding electrode into the skin at the neck. PowerLab stimulator delivered a 200-mV, 50-µs square-wave stimulus every 2 s. The stimulating electrode was inserted in the ankle near the Achilles tendon and subsequently into the sciatic notch at the hip, and M waves were recorded. The latency between Achilles tendon and sciatic notch was used to calculate nerve conduction velocity as described^64^. All measurements were made on coded animals by an observer unaware of the treatment groups.

### Corneal confocal imaging

Quantification of corneal nerves was performed in anaesthetized mice (using EZ Anesthesia Versaflex system, Braintree Scientific, Z-7150) using Retina Tomograph 3 with Rostock Cornea Module (Heidelberg Engineering) equipped with Tomocap (Heidelberg Engineering, 0220-001) as previously described^64^. In brief, lightly anaesthetized mice were transferred onto a small animal platform with anaesthesia maintained via a face mask. Forty sequential images of uniform magnification and size were collected and those containing nerves of the sub-basal plexus identified. ImageJ software (ImageJ 1.53e Java 1.8.0_172) was used to quantify corneal nerve area within each image, with data presented as pixels/image. All measurements were made on coded animals and images by an observer unaware of the treatment groups.

### Epidermal innervation

Quantification of epidermal innervation was performed in paw skin samples by immunostaining for the pan-neuronal protein PGP9.5, as described previously in detail^64^. In brief, paw skin samples were collected into 4% buffered paraformaldehyde (Thermo scientific, J19943-K2). Staining of epidermal nerves was performed using anti-PGP9.5 antibody (ProteinTech, 14730-1-AP; 1:500 dilution). Using 40× magnification of a light microscope, the number of PGP9.5-positive profiles present in the epidermis was calculated, length of skin section calculated, and IENF profile density expressed as profiles mm^−1^. All measurements were made on coded slides by an observer unaware of the treatment groups.

### Metabolite extraction and quantification

Plasma polar metabolites were extracted from 3 µl of plasma spiked with a known amount of ^13^C- and ^15^N-labelled standards (Cambridge Isotope Laboratories, MSK-A2-1.2). Tissue metabolite extraction was performed as described before^21^. In brief, ~20 mg of tissue was homogenized for 2 min using Precellys beads with 500 µl −20 °C methanol, 400 µl ice-cold saline and 100 µl ice-cold water and spiked with ^13^C/^15^N polar metabolite standards (Cambridge Isotope Laboratories, MSK-A2-1.2), 20 pmol of sphinganine-d7 (Avanti Polar Lipids, 860658), 2 pmol of deoxysphinganine-d3 (Avanti Polar Lipids, 860474), 100 pmol of ^13^C-dihydroceramide-d7 (Avanti Polar Lipids, 330726), 200 pmol of C_15_-ceramide-d7 (Avanti Polar Lipids, 860681), 10 pmol of d18:1-d7 glucosylsphingosine (Avanti Polar Lipids, 860695), 100 pmol of d18:1-d7/15:0 glucosylceramide (Avanti Polar Lipids, 330729), 100 pmol of d18:1-d7/15:0 lactosylceramide (Avanti Polar Lipids, 330727), 200 pmol of sphingosine-d7 (Avanti Polar Lipids, 860657), and 200 pmol of d18:1/18:1-d9 sphingomyelin (Avanti Polar Lipids, 791649). The identification of 1-deoxydihydroceramides was confirmed via retention time matching and analysis of m18:0/24:1 deoxyDHCer (Avanti Polar Lipids, 860464) and m18:0/16:0 deoxyDHCer (Avanti Polar Lipids, 860462) standards, and normalization for 1-deoxydihydroceramides was done with the 13C-dihydroceramide-d7 standard. Homogenate aliquot of 50 µl was taken to determine tissue protein content using BCA protein assay (Lambda Biotech, G1002). The remaining homogenate was transferred to a 2 ml Eppendorf tube and 1 ml of −20 °C chloroform was added. Samples were vortex-mixed for 5 min and spun down for 5 min at 4 °C at 15,000*g*. The organic phase was collected and 2 μl of formic acid was added to the remaining polar phase which was re-extracted with 1 ml of −20 °C chloroform. Combined organic phases were dried and the pellet was resuspended in 100 μl of buffer containing 100% methanol, 1 mM ammonium formate and 0.2% formic acid. Data represents ion counts normalized by class-specific internal standards and tissue protein content, with stacked plots to represent acyl-chain distribution.

#### Gas chromatography–mass spectrometry

Quantification of polar metabolites was determined after derivatization with 2% (w/v) methoxyamine hydrochloride (Thermo Scientific) in pyridine (37 °C for 60 min) and with *N*-tertbutyldimethylsilyl-*N*-methyltrifluoroacetamide (MTBSTFA) with 1% *tert*-butyldimethylchlorosilane (tBDMS) (Regis Technologies) (37 °C for 30 min). Polar derivatives were analysed by GC–MS using a DB-35MS column (30 m × 0.25 mm internal diameter × 0.25 μm, Agilent J&W Scientific) installed in an Agilent 7890 A gas chromatograph interfaced with an Agilent 5975 C mass spectrometer as previously described^65^. Plasma glucose enrichment was determined using propionic anhydride derivatization as previously described^66^. Natural isotope abundance was corrected using in-house script^51^.

#### Targeted sphingolipid quantification

Quantification of sphingolipid metabolites was determined using triple quadrupole liquid chromatography–mass spectrometry platform (Agilent 6460). Sphingolipid species were separated on a C8 column (Spectra 3 μm C8SR 150 × 3 mm inner diameter, Peeke Scientific) as previously described^67^. Mobile phase A was composed of 100% HPLC-grade water containing 2 mM ammonium formate and 0.2% formic acid and mobile phase B consisted of 100% methanol containing 0.2% formic acid and 1 mM ammonium formate. The flow rate was 0.5 ml min^−1^. The gradient elution programme consisted of the following profile: 0 min, 82% B; 3 min, 82% B; 4 min, 90% B, 18 min, 99% B; 25 min, 99%, 27 min, 82% B, 30 min, 82% B. Column re-equilibration followed each sample and lasted 10 min. The capillary voltage was set to 3.5 kV, the drying gas temperature was 350 °C, the drying gas flow rate was 10 l min^−1^, and the nebulizer pressure was 60 psi. Sphingolipid species were analysed by selective reaction monitoring (SRM) of the transition from precursor to product ions at associated optimized collision energies and fragmentor voltages (Supplementary Table 2). Quantification of sphingolipid species was performed using spiked-in deuterated standards.

#### High-resolution LC–MS/MS of polar metabolites

Around 10–20 mg of frozen tissue was extracted with 800 µl of −20 °C 5:3:2 acetonitrile: methanol:water solution spike with a known concentration of norvaline as an internal standard using the Precellys Evolution Homogenizer (Bertin Technologies)^68^. After extraction, a 50-µl aliquot was taken for protein quantification using BCA protein assay (Lambda Biotech, G1002), and the remaining extract was spun for 10 min at 21,000*g* at 4 °C. The supernatant was then transferred into a glass vial, and chromatographic separation and compound identification performed using Q Exactive Orbitrap MS with a Vanquish Flex Binary UHPLC system (ThermoFisher Scientific) on an iHILIC-(P) Classic, 150 mm by 2.1 mm, 5-mm particle, 200-Å (Hilicon) column at 45 °C. Chromatography was performed using a gradient of 20 mM ammonium carbonate, adjusted to pH 9.4 with 0.1% ammonium hydroxide (25%) solution (mobile phase A) and 100% acetonitrile (mobile phase B), both at a flow rate of 0.2 ml min^−1^. The liquid chromatography gradient ran linearly from 80 to 20% B from 2 to 17 min and then from 20 to 80% B from 17 to 18 min and then held at 80% B from 18 to 25 min.

#### High-resolution LC–MS/MS of lipids

Liver samples were extracted in 400 µl of −20 °C methanol using the Precellys Evolution Homogenizer (Bertin Technologies) spiked with EquiSPLASH labelled standard (Avanti Polar Lipids, 330731) and norvaline. After extraction, 50 µl aliquot was taken to quantify protein content using BCA protein assay (Lambda Biotech, G1002), and to the remaining extract were added 400 µl of −20 °C chloroform and 400 µl of ice-cold water. After vortexing for 5 min, samples were spun for 5 min at 4 °C at 15,000*g*, and the organic phase was collected. Two microlitres of formic acid were added to the remaining polar phase which was re-extracted with 400 µl of −20 °C chloroform, samples were vortex-mixed, and spun as described above. Combined organic phases were dried and the pellet was resuspended in 100 μl of isopropanol.

Chromatographic separation and lipid species identification was performed using Q Exactive orbitrap mass spectrometer with a Vanquish Flex Binary UHPLC system (Thermo Scientific) equipped with an Accucore C30, 150 × 2.1 mm, 2.6 µm particle (Thermo) column at 40 °C. Five microlitres of sample was injected. Chromatography was performed using a gradient of 40:60 v/v water: acetonitrile with 10 mM ammonium formate and 0.1% formic acid (mobile phase A) and 10:90 v/v acetonitrile: propan-2-ol with 10 mM ammonium formate and 0.1% formic acid (mobile phase B), both at a flow rate of 0.2 ml min^−1^. The liquid chromatography gradient ran from 30% to 43% B from 3–8 min, then from 43% to 50% B from 8-9 min, then 50–90% B from 9–18 min, then 90–99% B from 18–26 min, then held at 99% B from 26–30 min, before returning to 30% B in 6 min and held for a further 4 min.

Lipids were analysed in positive mode using spray voltage 3.2 kV. Sweep gas flow was 1 arbitrary units, auxiliary gas flow 2 arbitrary units and sheath gas flow 40 arbitrary units, with a capillary temperature of 325 °C. Full mass spectrometry (scan range 200–2,000 *m*/*z*) was used at 70,000 resolution with 10^6^ automatic gain control and a maximum injection time of 100 ms. Data dependent MS2 (Top 6) mode at 17,500 resolution with automatic gain control set at 10^5^ with a maximum injection time of 50 ms was used. Data were analysed using EI-Maven software, and peaks normalized to Avanti EquiSPLASH internal standard. Lipid species specific fragments used for identification and quantification are presented in the Supplementary Table 3.

#### Plasma sphingoid base extraction, hydrolysis and LC–MS analysis

Plasma sphingolipids were processed as previously described with minor modifications^69^. In brief, 50 µl of plasma was mixed with 0.5 ml of methanol and spiked with internal standards, sphinganine-d7, sphingosine-d7 and deoxysphinganine-d3 (Avanti lipids). The samples were placed on a mixer for 1 h at 37 °C, centrifuged at 2,800*g* and the supernatant collected and acid hydrolysed overnight at 65 °C with 75 µl of methanolic HCl (1N HCl,10M H_2_O in methanol). Next, 100 µl of 10 M KOH was added to neutralize. 625 µl of chloroform, 100 µl of 2N NH_4_OH and 500 µl of alkaline water were added, samples vortex-mixed and centrifuged for 5 min at 16,000*g*. The lower organic phase was washed three times with alkaline water and dried under air. LC–MS analysis was performed on an Agilent 6460 QQQ LC–MS/MS. Metabolite separation was achieved with a C18 column (Luna 100 × 2.1 mm, 3 µm, Phenomenex). Mobile phase A was composed of a 60:40 ratio of methanol:water and mobile phase B consisted of 100% methanol with 0.1% formic acid with 5 mM ammonium formate added to both mobile phases. The gradient elution programme consisted of holding at 40% B for 0.5 min, linearly increasing to 100% B over 15 min, and maintaining it for 9 min, followed by re-equilibration to the initial condition for 10 min. The capillary voltage was set to 3.5 kV, the drying gas temperature was 350 °C, the drying gas flow rate was 10 l min^−1^, and the nebulizer pressure was 60 psi. Sphingoid bases were analysed by SRM of the transition from precursor to product ions at associated optimized collision energies and fragmentor voltages^16^. Sphingoid bases were then quantified from spiked internal standards of known concentration.

### Serine dehydratase activity assay

Frozen liver and kidney samples were extracted in an ice-cold buffer containing 50 mM KH_2_PO_4_, 1 mM Na_2_EDTA, and 1mM DTT, pH 8.0 using glass homogenizer. Maximal enzyme activity was determined using coupled-enzyme reaction with lactate dehydrogenase (Sigma 10127230001) in the presence of 200 mM serine, 0.25 mM NADH, 0.17 mM pyridoxal phosphate, and 1 mM DTT for 3 min. Tissue homogenate protein quantification was subsequently determined using BCA protein assay (Lambda Biotech, G1002), and maximal enzyme activity expressed in international units (U) per mg of protein.

### Gene expression analysis

RNA was extracted from ~20 mg of liver tissue using Direct-Zol RNA kit (Direct-Zol RNA Miniprep Plus kit, Zymo Research) according to the manufacturer’s instructions. cDNA synthesis was performed using iScript Reverse Transcription Supermix for RT–PCR (iScript Reverse Transcription Supermix, Bio-Rad) according to the manufacturer’s instructions using the following protocol: 5 min at 25 °C, 20 min 46 °C, 1 min 95 °C. PCR reactions were carried out using 96-well plates on an Applied Biosystems ViiA 7 Real-Time PCR System using the following parameters: 95 °C for 20 s, 40 cycles of 95 °C for 1 s, and 60 °C for 20 s. The final volume (10 µl) of PCR SYBR-Green reaction consisted of 5 µl fast SYBR-Green Master Mix (Applied Biosystems), 2 µl cDNA, 1 µl of 5 µM forward and reverse primers, and 1 µl of water.

Primers used are as follows. 18s forward: AGTCCCTGCCCTTTGTACACA, 18s reverse: CGATCCGAGGGCCTCACTA; *Acc1* forward: AATGAACGTGCAATCCGATTTG, *Acc1* reverse: ACTCCACATTTGCGTAATTGTTG; *Acc2* forward: CGCTCACCAACAGTAAGGTGG, *Acc2* reverse: GCTTGGCAGGGAGTTCCTC; *Acly* forward: AATCCTGGCTAAAACCTCGCC, *Acly* reverse: GCATAGATGCACACGTAGAACT; actin forward: GGCTGTATTCCCTCCATCG, actin reverse: CCAGTTGGTAACAATGCCATGT; *Aldh1l1* forward: AGCCACCTATGAGGGCATTC, *Aldh1l1* reverse: TGAGTGTCGAGTTGAAAAACGTC; *Aldh1l2* forward: ACCAGCCGGGTTTATTTCAAA, *Aldh1l2* reverse: ACTCCCACTACTCGGTGGC; *Dgat1* forward: CTGATCCTGAGTAATGCAAGGTT, *Dgat1* reverse: TGGATGCAATAATCACGCATGG; *Dgat2* forward: GCGCTACTTCCGAGACTACTT, *Dgat2* reverse: GGGCCTTATGCCAGGAAACT; *Dhcr7* forward: AGGCTGGATCTCAAGGACAAT, *Dhcr7* reverse: GCCAGACTAGCATGGCCTG; *Dhcr24* forward: CTCTGGGTGCGAGTGAAGG, *Dhcr24* reverse: TTCCCGGACCTGTTTCTGGAT; *Dld* forward: AGCTGGAGTCGTGTGTACC, *Dld* reverse: GAACCTATCACTGTCACGTCA; *Fasn* forward: GGAGGTGGTGATAGCCGGTAT, *Fasn* reverse: TGGGTAATCCATAGAGCCCAG; *Fdft1* forward: GTTTGAAGACCCCATAGTTGGTG, *Fdft1* reverse: CACATCTACGTTCTCTGGCTTAG; *Fdps* forward: GGAGGTCCTAGAGTACAATGCC, *Fdps* reverse: AAGCCTGGAGCAGTTCTACAC; *Ggps1* forward: TTCACAGGCATTTAATCACTGGC, *Ggps1* reverse: ACCACGTCGGAGCTTTGAAC; *Gldc* forward: CTCCTGCCCAGACACGAT, *Gldc* reverse: GGGACCGTCTTCTCGATGAG; *Gpat1* forward: CTTGGCCGATGTAAACACACC, *Gpat1* reverse: CTTCCGGCTCATAAGGCTCTC; *Gpat4* forward: TCAAAGAAATTCGTCGAAGTGGT, *Gpat4* reverse: CCTTTCCGGCAAAAGTAGAAGAT; *Hmgcs1* forward: AACTGGTGCAGAAATCTCTAGC, *Hmgcs1* reverse: GGTTGAATAGCTCAGAACTAGCC; *Hmgcr* forward: AGCTTGCCCGAATTGTATGTG, *Hmgcr* reverse: TCTGTTGTGAACCATGTGACTTC; *Lss* forward: TCGTGGGGGACCCTATAAAAC, *Lss* reverse: CGTCCTCCGCTTGATAATAAGTC; *Mthfd1* forward: CTCCTGTCCCAAGTGACATTG, *Mthfd1* reverse: TAGCCTTCGTTTCCCCGTACA; *Mthfd2* forward: AGTGCGAAATGAAGCCGTTG, *Mthfd2* reverse: GACTGGCGGGATTGTCACC; *Mthfd1l* forward: GCATGGCCTTACCCTTCAGAT, *Mthfd1l* reverse: GTACGAGCTTCCCCAGATTGA; *Mthfd2l* forward: AAGGACGTTGATGGATTTCACAT, *Mthfd2l* reverse: GATGATTTCCCAAACGGCACT; *Mthfr* forward: AGATGAGGCGCAGAATGGAC, *Mthfr* reverse: CATCCGGTCAAACCTGGAGAT; *Mtr* forward: TCCTCCTCGGCCTATCTTTATTT, *Mtr* reverse: GGTCCGAATGAGACACGCT; *Mvk* forward: GGTGTGGTCGGAACTTCCC, *Mvk* reverse: CCTTGAGCGGGTTGGAGAC; *Mvd* forward: ATGGCCTCAGAAAAGCCTCAG, *Mvd* reverse: TGGTCGTTTTTAGCTGGTCCT; *Pmvk* forward: CCTATGGGGCTGTGATACAGA, *Pmvk* reverse: TCTCCGTGGTTCTCAATGACC; *Psat* forward: CAGTGGAGCGCCAGAATAGAA, *Psat* reverse: CCTGTGCCCCTTCAAGGA; *Psph* forward: TGAGTACGCAGGTTTTGATGAG, *Psph* reverse: TGAGTACGCAGGTTTTGATGAG; *Phgdh* forward: ATGGCCTTCGCAAATCTGC, *Phgdh* reverse: AGTTCAGCTATCAGCTCCTCC; *Scd1* forward: TTCTTGCGATACACTCTGGTGC, *Scd2* reverse: CGGGATTGAATGTTCTTGTCGT; *Scd2* forward: GATCTCTGGCGCTTACTCAGC, *Scd2* reverse: CTCCCCAGTGGTGAGAACTC; *Sds* forward: GAAGACCCCACTTCGTGACAG, *Sds* reverse: TCTTGCAGAGATGCCCAATGC; *Shmt1* forward: CAGGGCTCTGCTTGATGCAC, *Shmt1* reverse: CGTAACGCGCTCTTGTCAC; *Shmt2* forward: TGGCAAGAGATACTACGGAGG, *Shmt2* reverse: AGATCCGCTTGACATCAGACA; *Sqle* forward: ATAAGAAATGCGGGGATGTCAC, *Sqle* reverse: ATATCCGAGAAGGCAGCGAAC; *Srebp1a* forward: TAGTCCGAAGCCGGGTGGGCGCCGG, *Srebp1a* reverse: GATGTCGTTCAAAACCGCTGTGTGTC; *Srebp1c* forward: AAGCAAATCACTGAAGGACCTGG, *Srebp1c* reverse: AAAGACAAGCTACTCTGGGAG; *Srebp2* forward: GGATCCTCCCAAAGAAGGAG, *Srebp2* reverse: TTCCTCAGAACGCCAGACTT; *Tyms* forward: GGAAGGGTGTTTTGGAGGAGT, *Tyms* reverse: GCTGTCCAGAAAATCTCGGGA.

### Western blotting

To compare tissue protein levels, ~20 mg of tissue was homogenized in RIPA buffer supplemented with 5 mM EDTA solution (Thermo Scientific), protease inhibitor cocktail (cOmplete, Roche) and phosphatase inhibitor cocktail (PhosSTOP, Roche), and placed on ice for 30 min. Tissue lysate was centrifuged at 13,000*g* for 10 min at 4 °C, and the supernatant was stored at −80 °C. Homogenate protein content was determined using the bicinchoninic acid assay (Thermo Scientific). Protein samples were prepared in a Laemmli buffer (NuPAGE LDS Sample Buffer, Life Technologies), and were run a 4–15% precast gel (Mini-PROTEAN TGX, Bio-Rad) for 2 h at constant 100 V and transferred on a polyvinylidenedifluoride (PVDF) membrane for 2 h at constant 250 mA in an ice-chilled transfer tank. The membrane was blocked with 5% milk and incubated overnight at 4 °C with a primary antibody against ACLY (Cell Signaling 13390, 1:1,000), ACC (Cell Signaling 3662, 1:2,000), p-AKT Ser473 (Cell Signaling 9271, 1:1,000), p-AKT Ser308 (Cell Signaling 9275, 1:1,000), AKT (Cell Signaling 75692, 1:1,000), SCD1 (Cell Signaling 2794, 1:1,000), GAPDH (Cell Signaling 5174, 1:4,000), and vinculin (Cell Signaling 4650, 1:1,000). After washing with TBS-T, membranes were incubated with a horseradish peroxidase-conjugated secondary antibody (Cell Signaling 7074, 1:5,000) for 1 h at room temperature and incubated with enhanced chemiluminescence liquid (Clarity Western ECL Substrate, Bio-Rad) for 5 min. Densitometry quantification was performed using Image Lab software (Bio-Rad). For raw scans with molecular weight markers, see Supplementary Fig. 1.

### Statistical analysis

Data are expressed as mean ± s.e.m. unless stated otherwise. Statistical analysis was performed with Prism software (GraphPad Prism 9.3.1) using two-sided independent *t*-test to compare two groups, one-way ANOVA with Fisher’s least significant difference post hoc test to compare more than two groups, two-way ANOVA with Fisher’s least significant difference post hoc test to compare two-factor study design, and PERMANOVA analysis to explore RPCA plots. For all tests, *P* < 0.05 was considered significant. All data points in the manuscript represent individual biological replicates. No statistical methods were used to predetermine sample size.

### Reporting summary

Further information on research design is available in the Nature Portfolio Reporting Summary linked to this article.

## Online content

Any methods, additional references, Nature Portfolio reporting summaries, source data, extended data, supplementary information, acknowledgements, peer review information; details of author contributions and competing interests; and statements of data and code availability are available at 10.1038/s41586-022-05637-6.

## Supplementary information

Supplementary Figure 1 Supplementary Fig. 1 contains the raw western blot images.

Reporting Summary

Supplementary Table 1 Dietary composition.

Supplementary Table 2 LC–MS/MS transitions.

Supplementary Table 3 High-resolution mass spectrometry transitions.

## Data Availability

Source data for microbiome algorithms and immunoblots are provided as Supplementary Information. The whole-microbiome genome sequencing raw data was uploaded to Qiita^53^, where we followed the default processing workflow. High-resolution and targeted mass spectrometry data data is available at the NIH Common Fund’s National Metabolomics Data Repository (NMDR) website, the Metabolomics Workbench, https://www.metabolomicsworkbench.org where it has been assigned Project ID M8JD81 (10.21228/M8JD81). The data can be accessed directly via the Project ID M8JD81. Additional data that support the findings are available from the corresponding author upon reasonable request.
