# Supplementary material for: Insulin-regulated serine and lipid metabolism drive peripheral neuropathy
Source: Nature. 2023 Jan 25;614(7946):118–24. doi: 10.1038/s41586-022-05637-6 (PMC9891999; doi:10.1038/s41586-022-05637-6)

nature portfolio

https://doi.org/10.1038/s41586-022-05637-6

Supplementary information

Insulin-regulated serine and lipid metabolism drive peripheral neuropathy

In the format provided by the authors and unedited

Supplementary Figure 1 (raw data gels) corresponding to Extended Data Fig. 2s.

ACLY

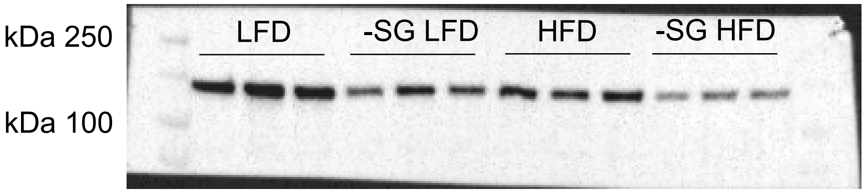

GAPDH

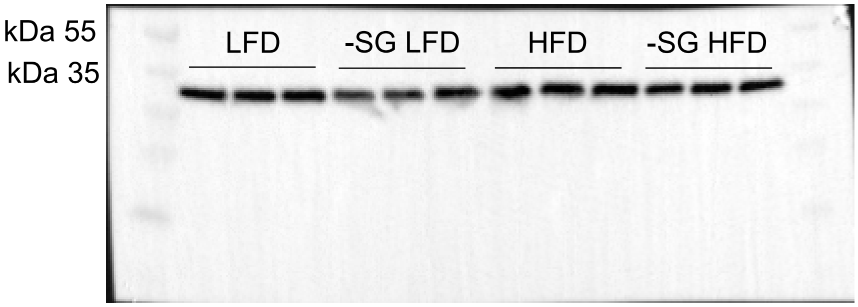

ACC

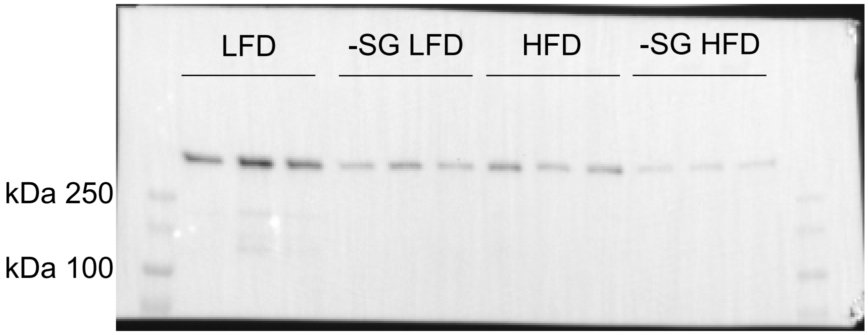

GAPDH

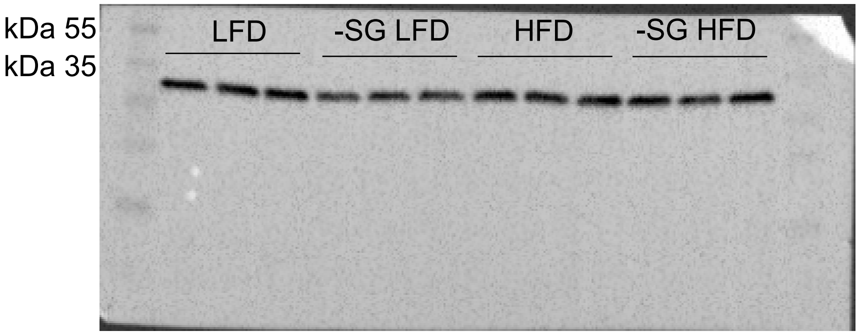

SCD1

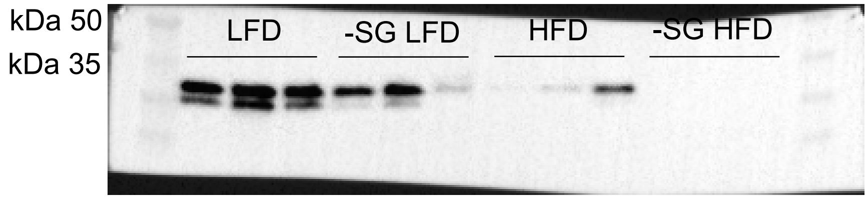

Vinculin

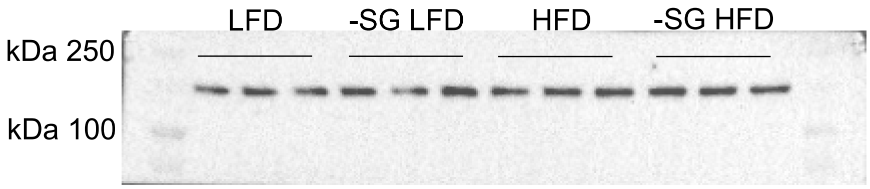

P-Akt[S473]

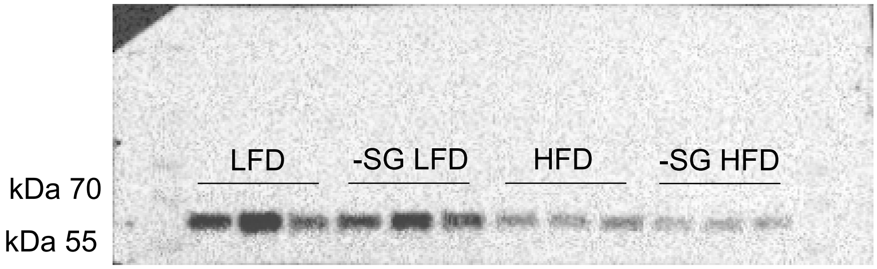

P-Akt[T308]

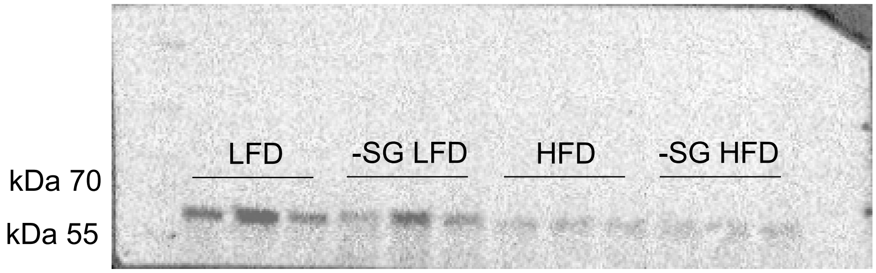

Total Akt

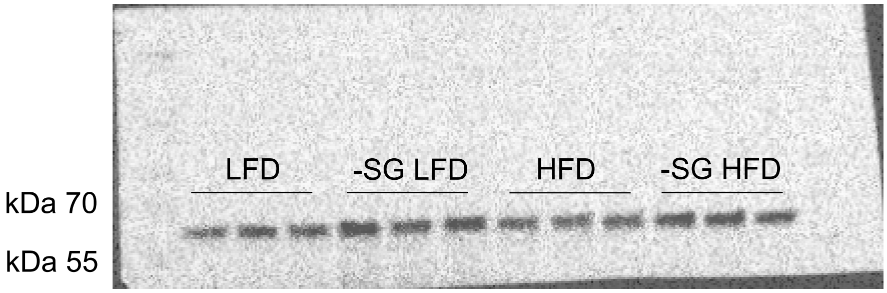

GAPDH

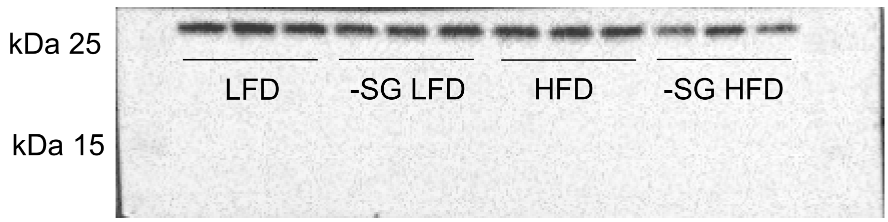

Supplement: Supplementary file 1 — Supplementary Fig. 1 contains the raw western blot images. [file 41586_2022_5637_MOESM1_ESM.pdf]
