# Supplementary material for: Insulin-regulated serine and lipid metabolism drive peripheral neuropathy
Source: Nature. 2023 Jan 25;614(7946):118–24. doi: 10.1038/s41586-022-05637-6 (PMC9891999; doi:10.1038/s41586-022-05637-6)
Supplement: Supplementary file 2 — Reporting Summary [file 41586_2022_5637_MOESM2_ESM.pdf]

nature portfolio

Corresponding author(s): Christian Metallo

Last updated by author(s): Nov 17, 2022

Reporting Summary

Nature Portfolio wishes to improve the reproducibility of the work that we publish. This form provides structure for consistency and transparency in reporting. For further information on Nature Portfolio policies, see our Editorial Policies and the Editorial Policy Checklist.

Statistics

For all statistical analyses, confirm that the following items are present in the figure legend, table legend, main text, or Methods section.

n/a	Confirmed	
☐	☒	The exact sample size (*n*) for each experimental group/condition, given as a discrete number and unit of measurement
☐	☒	A statement on whether measurements were taken from distinct samples or whether the same sample was measured repeatedly
☐	☒	The statistical test(s) used AND whether they are one- or two-sided<br>*Only common tests should be described solely by name; describe more complex techniques in the Methods section.*
☒	☐	A description of all covariates tested
☐	☒	A description of any assumptions or corrections, such as tests of normality and adjustment for multiple comparisons
☐	☒	A full description of the statistical parameters including central tendency (e.g. means) or other basic estimates (e.g. regression coefficient) AND variation (e.g. standard deviation) or associated estimates of uncertainty (e.g. confidence intervals)
☐	☒	For null hypothesis testing, the test statistic (e.g. *F*, *t*, *r*) with confidence intervals, effect sizes, degrees of freedom and *P* value noted<br>*Give P values as exact values whenever suitable.*
☒	☐	For Bayesian analysis, information on the choice of priors and Markov chain Monte Carlo settings
☐	☒	For hierarchical and complex designs, identification of the appropriate level for tests and full reporting of outcomes
☒	☐	Estimates of effect sizes (e.g. Cohen's *d*, Pearson's *r*), indicating how they were calculated

Our web collection on statistics for biologists contains articles on many of the points above.

Software and code

Policy information about availability of computer code

Data collection	Confocal corneal imaging was performed using Retina Tomograph 3 with Rostock Cornea Module (Heidelberg Engineering Inc.) equipped with Tomocap (Heidelberg Engineering, cat. no. 0220-001).
Data analysis	All software is commercially or freely available, literature references are included in the Method section. Mass spectrometry data were processed using using AMDIS, El-Maven v0.11.0, and Agilent Mass Hunter. Individual figure panels were prepared using GraphPad Prism 9.3.1 and Adobe Illustrator 24.3. Quantification of corneal nerves was performed using ImageJ software (ImageJ 1.53e Java 1.8.0_172).<br><br>The whole microbiome genome sequencing raw data was uploaded to Qiita, where we followed their default processing workflow. In summary, the raw reads were adaptor filtered using the auto-detect parameters in fastp version 2063 and host (mouse) filtered using minimap2 version 2.1764. The resulting sequences were aligned using Bowtie 2 version 2.4.265 to the Web of Life (WoL) reference database66 via the Web of Life Toolkit App (https://github.com/qiyunzhu/woltka); this step generated tables at genus, species, per genome, and per gene tables. For all analyzes we used the per genome table; then for alpha diversity we removed any samples below 1,273,062 sequences per sample and for beta diversity analysis we rarefied at the same value. Downstream analyses were performed in QIIME 2 version 2020.116. To asses global microbiota alterations, alpha diversity analysis was performed through Faith's PD68 and beta diversity through robust principal-component analysis (RPCA)69 and resulting Aitchison distances were evaluated through permutational multivariate analysis of variance (PERMANOVA).

For manuscripts utilizing custom algorithms or software that are central to the research but not yet described in published literature, software must be made available to editors and reviewers. We strongly encourage code deposition in a community repository (e.g. GitHub). See the Nature Portfolio guidelines for submitting code & software for further information.

Data

Policy information about availability of data

All manuscripts must include a data availability statement. This statement should provide the following information, where applicable:

- Accession codes, unique identifiers, or web links for publicly available datasets
- A description of any restrictions on data availability
- For clinical datasets or third party data, please ensure that the statement adheres to our policy

Source data for microbiome algorithms and immunoblots are provided as Supplementary Information (Supplementary Figure 1). The whole microbiome genome sequencing raw data was uploaded to Qiita, where we followed their default processing workflow. The resulting sequences were aligned using Bowtie 2 version 2.4.25 to the Web of Life (WoL) reference database (Zhu et al Nat. Commun. 10, 5477, doi:10.1038/s41467-019-13443-4) via the Web of Life Toolkit App (https://github.com/qiyunzhu/woltka).

High-resolution and targeted mass spectrometry data data is available at the NIH Common Fund's National Metabolomics Data Repository (NMDR) website, the Metabolomics Workbench, https://www.metabolomicsworkbench.org where it has been assigned Project ID (http://dx.doi.org/10.21228/M8JD81). The data can be accessed directly via it's Project DOI: (http://dx.doi.org/10.21228/M8JD81). This work is supported by NIH grant U2C-DK119886.

Additional data that support findings is available from the corresponding author upon reasonable request.

Field-specific reporting

Please select the one below that is the best fit for your research. If you are not sure, read the appropriate sections before making your selection.

☒ Life sciences ☐ Behavioural & social sciences ☐ Ecological, evolutionary & environmental sciences

For a reference copy of the document with all sections, see nature.com/documents/nr-reporting-summary-flat.pdf

Life sciences study design

All studies must disclose on these points even when the disclosure is negative.

Sample size	No statistical methods were used to predetermine sample size. Sample sizes were determined based on our and other investigators experience with the respective animal studies (Wallace et al. Nat Chem Biol 2018 and Calcutt et al. JCI 2017). The sample sizes were found to be adequate based on the magnitude and consistency of measurable differences between groups.
Data exclusions	No data were excluded.
Replication	All replicate experiments were successful. All experimental details necessary to replicate the results in other laboratories were provided in the Methods section. At least 3 independent biological replicates were performed in each condition.
Randomization	Sex- and age-matched mice were randomly assigned to individual dietary groups ensuring there were no baseline differences in body weight.
Blinding	The investigators were blinded to experimental conditions including paw skin sectioning, and IENF and tactile sensing quantification. The data reported for the metabolic and metabolomics experiments were based on quantitative cellular and metabolic measurements that are not subject to biases. In this context, the investigators were not blinded to group allocation, tissue collection, and processing for metabolomics or transcriptomics analysis.

Reporting for specific materials, systems and methods

We require information from authors about some types of materials, experimental systems and methods used in many studies. Here, indicate whether each material, system or method listed is relevant to your study. If you are not sure if a list item applies to your research, read the appropriate section before selecting a response.

Materials & experimental systems

n/a	Involved in the study	
☐	☒	Antibodies
☒	☐	Eukaryotic cell lines
☒	☐	Palaeontology and archaeology
☐	☒	Animals and other organisms
☒	☐	Human research participants
☒	☐	Clinical data
☒	☐	Dual use research of concern

Methods

n/a	Involved in the study	
☒	☐	ChIP-seq
☒	☐	Flow cytometry
☒	☐	MRI-based neuroimaging

Antibodies

Antibodies used	ACLY (Cell Signaling #13390, 1:1000), ACC (Cell Signaling #3662, 1:2000), P-AktSer473 (Cell Signaling #9271, 1:1000), P-AktSer308 (Cell Signaling #9275, 1:1000), Akt (Cell Signaling #75692, 1:1000), SCD1 (Cell Signaling #2794, 1:1000), GAPDH (Cell Signaling #5174, 1:4000), vinculin (Cell Signaling #4650, 1:1000), anti-rabbit IgG, HRP-linked Antibody (Cell Signaling #7074, 1:5000), and rabbit PGP9.5 antibody (Proteintech Cat# 14730-1-AP; 1:500)
Validation	The manufacturer provided a validation certificate for all the antibodies used for Western applications. Antibodies for western blotting were validated for human reactivity by the manufacturer and used per their instructions. Additional information on validation (publication, certificate, manufacturer western blot) can be found on the manufacturers' websites listed below:<br><br>- ACLY (Cell Signaling #13390) https://www.cellsignal.com/products/primary-antibodies/atp-citrate-lyase-d1x6p-rabbit-mab/13390<br>- ACC (Cell Signaling #3662) https://www.cellsignal.com/products/primary-antibodies/acetyl-coa-carboxylase-antibody/3662<br>- P-AktSer473 (Cell Signaling #9271) https://www.cellsignal.com/products/primary-antibodies/phospho-akt-ser473-antibody/9271<br>- P-AktSer308 (Cell Signaling #9275) https://www.cellsignal.com/products/primary-antibodies/phospho-akt-thr308-antibody/9275<br>- Akt (Cell Signaling# 75692) https://www.cellsignal.com/products/primary-antibodies/akt1-d9r8k-rabbit-mab/75692<br>- SCDl (Cell Signaling #2794) https://www.cellsignal.com/products/primary-antibodies/scd1-c12h5-rabbit-mab/2794<br>- GAP DH (Cell Signaling #5174) https://www.cellsignal.com/products/primary-antibodies/gapdh-d16h11-xp-rabbit-mab/5174<br>- vinculin (Cell Signaling #4650) https://www.cellsignal.com/products/primary-antibodies/vinculin-antibody/4650<br>- anti-rabbit IgG, HRP-linked Antibody (Cell Signaling #7074) https://www.cellsignal.com/products/secondary-antibodies/anti-rabbit-igg-hrp-linked-antibody/7074<br>- PGP9.5 antibody (Proteintech Cat# 14730-1-AP; 1:500) https://www.ptglab.com/products/UCHL1-Antibody-14730-1-AP.htm

Animals and other organisms

Policy information about studies involving animals; ARRIVE guidelines recommended for reporting animal research

Laboratory animals	Ten- to sixteen-week-old C57BL/6J (JAX# 000664) or BKS-db/db mice (JAX #000642) were used in this manuscript.
Wild animals	No wild animals were used in this manuscript.
Field-collected samples	This manuscript did not involve field-collected samples.
Ethics oversight	All animal experiments were approved and conducted in accordance with the Institutional Animal Care and Use Committee (IACUC) of the University of California San Diego and the Salk Institute.

Note that full information on the approval of the study protocol must also be provided in the manuscript.
